# Metallic WO_2_-Promoted CoWO_4_/WO_2_ Heterojunction with Intercalation-Mediated Catalysis for Lithium–Sulfur Batteries

**DOI:** 10.1007/s40820-025-01849-3

**Published:** 2025-07-18

**Authors:** Chan Wang, Pengfei Zhang, Jiatong Li, Rui Wang, Changheng Yang, Fushuai Yu, Xuening Zhao, Kaichen Zhao, Xiaoyan Zheng, Huigang Zhang, Tao Yang

**Affiliations:** 1 Shaanxi Key Laboratory for Theoretical Physics Frontiers, Institute of Modern Physics, Northwest University, Xi’an, 710127 People’s Republic of China; 2 Shaanxi Key Laboratory of Degradable Biomedical Materials, School of Chemical and Engineering, Institute of Low-Carbon Technology Application, Northwest University, Xi’an, 710069 People’s Republic of China; 3 State Key Laboratory of Mesoscience and Engineering, Institute of Process Engineering, Chinese Academy of Sciences, Beijing, 100190 People’s Republic of China; 4 School of Chemical Engineering, University of the Chinese Academy of Sciences, No. 19(A) Yuquan Road, Shijingshan District, Beijing, 100049 People’s Republic of China

**Keywords:** Lithium sulfur batteries; Catalysis; Shuttle effect; Heterojunction

## Abstract

Lithium–sulfur (Li–S) batteries require efficient catalysts to accelerate polysulfide conversion and mitigate the shuttle effect. However, the rational design of catalysts remains challenging due to the lack of a systematic strategy that rationally optimizes electronic structures and mesoscale transport properties. In this work, we propose an autogenously transformed CoWO_4_/WO_2_ heterojunction catalyst, integrating a strong polysulfide-adsorbing intercalation catalyst with a metallic-phase promoter for enhanced activity. CoWO_4_ effectively captures polysulfides, while the CoWO_4_/WO_2_ interface facilitates their S–S bond activation on heterogenous catalytic sites. Benefiting from its directional intercalation channels, CoWO_4_ not only serves as a dynamic Li-ion reservoir but also provides continuous and direct pathways for rapid Li-ion transport. Such synergistic interactions across the heterojunction interfaces enhance the catalytic activity of the composite. As a result, the CoWO_4_/WO_2_ heterostructure demonstrates significantly enhanced catalytic performance, delivering a high capacity of 1262 mAh g^−1^ at 0.1 C. Furthermore, its rate capability and high sulfur loading performance are markedly improved, surpassing the limitations of its single-component counterparts. This study provides new insights into the catalytic mechanisms governing Li–S chemistry and offers a promising strategy for the rational design of high-performance Li–S battery catalysts.

**Supplementary Information:**

The online version contains supplementary material available at 10.1007/s40820-025-01849-3.

## Highlights

- The CoWO_4_/WO_2_ heterojunction was successfully constructed through hydrothermal synthesis of precursors followed by autogenous transformation induced by hydrogen reduction.
- The synergistic effect of CoWO_4_ and WO_2_ promotes the catalytic conversion of polysulfides and suppresses the shuttle effect.
- The CoWO_4_/WO_2_ heterojunction demonstrates significantly enhanced catalytic performance, delivering a high capacity of 1262 mAh g^−1^ at 0.1 C.

**Supplementary Information:**

The online version contains supplementary material available at 10.1007/s40820-025-01849-3.

## Graphical abstract

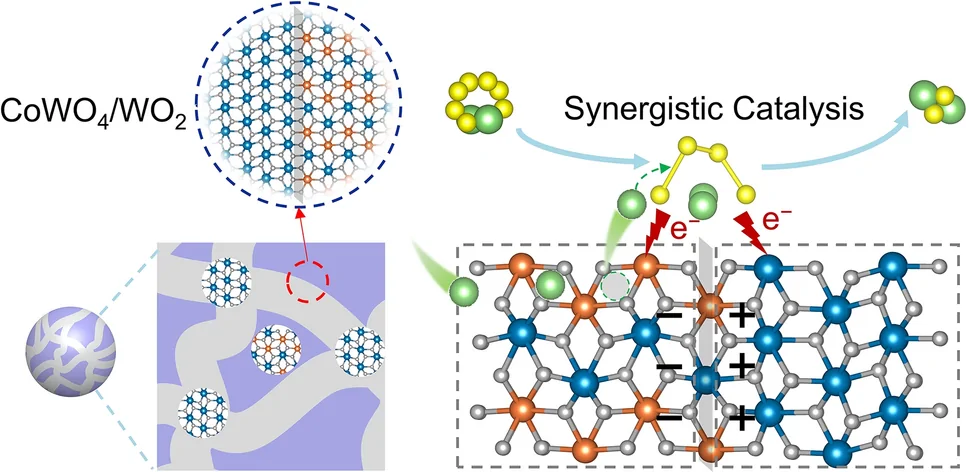

## Introduction

Lithium–sulfur (Li–S) batteries have garnered significant attention due to their exceptionally high theoretical specific capacity of 1675 mAh g^−1^ and a gravimetric energy density of 2600 Wh kg^−1^, as well as cost-effectiveness and environmental friendliness [1, 2]. However, the practical application of these advantages is fundamentally constrained by two interrelated challenges: the dissolution-induced shuttle effect of lithium polysulfides (LiPSs) and sluggish sulfur redox kinetics [3, 4]. These phenomena lead to poor cycling performance through active material loss and reaction inefficiency, particularly under high sulfur loading or elevated current density conditions [5].

To address these inherent limitations, current research efforts focus on developing multifunctional host architectures that integrate efficient LiPSs chemisorption with catalytic conversion, thereby suppressing shuttle behavior and enhancing redox kinetics [6–10]. Transition metal compounds have demonstrated particular promise in this regard, though their performance remains constrained by inherent material limitations [11]. For example, metal oxides (CoO [12], WO_3−x_ [13], W_18_O_49_ [14]) exhibit exceptional LiPSs adsorption through strong Lewis acid–base interactions and Coulombic effects, yet suffering from their intrinsically low electrical conductivity that severely constrains electrocatalytic performance. Conversely, chalcogenides (sulfides–selenides) [15–23], phosphides [24–28], and nitrides [29–32] possess good electron conduction and charge transfer capabilities but exhibit insufficient adsorption strength for effective LiPSs immobilization. These multiple requirements for adsorption capacity and catalytic activity underlines the critical need for innovative material designs that transcend conventional single-phase limitations.

Heterostructure engineering has emerged as an effective strategy to reconcile these competing requirements [33–37]. By creating interfacial electric fields and optimizing phase boundaries, materials such as CoSe_2_/Co_3_O_4_ and TiO_2_–TiN hybrids show enhanced charge redistribution that promotes both LiPSs adsorption and catalytic conversion [38, 39]. Nevertheless, conventional heterojunctions often neglect the critical aspect of lithium-ion transport dynamics, particularly under high-rate conditions where ionic diffusion becomes the rate-limiting factor. Meanwhile, lithium storage materials (e.g., Mo_6_S_8_ [40], Nb_18_W_16_O_93_ [41]) offer fast Li-ion transport and have been investigated not only for immobilizing sulfur species but also for accelerating LiPSs conversion. This complex interplay of adsorption, catalysis, and ionic transport presents a formidable challenge in sulfur host design.

**Fig. 1:**
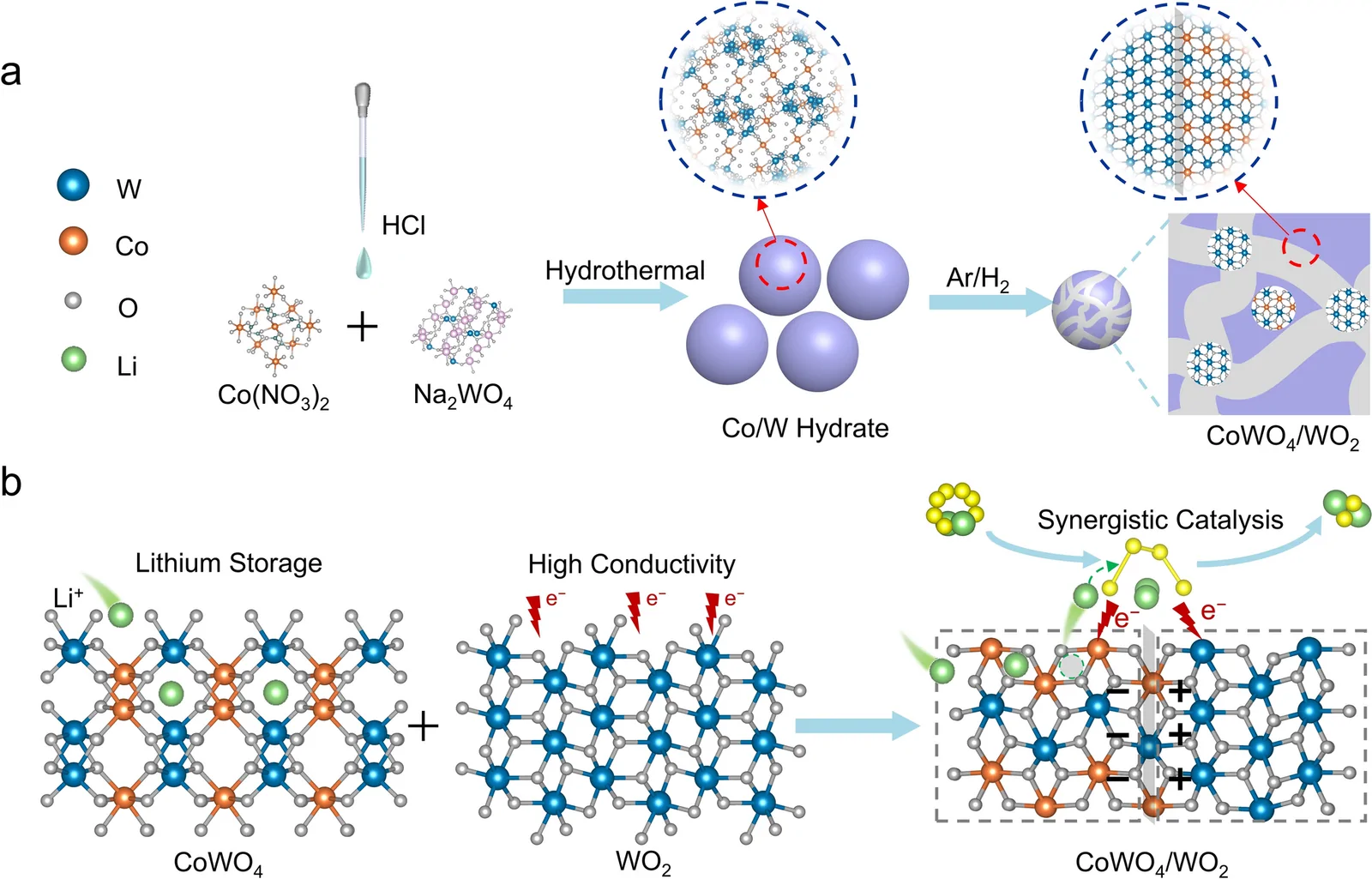
Schematical illustration of structural design and catalysis enhancement mechanism. **a** Synthesis of CoWO_4_/WO_2_ heterojunction catalysts via a hydrothermal method followed by H_2_ reduction. **b** Structural design and catalytic effects enabled by the CoWO_4_/WO_2_ heterojunction

In this work, we propose a rationally designed CoWO_4_/WO_2_ heterojunction catalyst synthesized via a hydrothermal route followed by an autogenous structural transformation induced by H_2_ reduction (Fig. 1a). The engineered CoWO_4_/WO_2_ interface unifies multiple functionalities unattainable by either component alone. CoWO_4_ strongly adsorbs LiPSs and interacts Li/S atoms through its partially filled d-orbitals, effectively weakening S–S bonds and lowering the activation barrier for LiPSs conversion. Moreover, Li-ion intercalation into CoWO_4_ within the Li–S voltage range forms a dynamic Li-ion reservoir, while vacant cation sites create direct pathways for rapid Li-ion transport. Simultaneously, the in situ-formed WO_2_ phase not only enhances the interfacial catalytic effect by donating electrons to CoWO_4_ owing to the difference in their work functions, but also provides a highly conductive electron highway by leveraging its metallic character. The synergistic interplay of these features enables superior catalytic performance compared to pristine CoWO_4_ or WO_2_ (Fig. 1b). Specifically, the CoWO_4_/WO_2_ heterojunction delivers a high specific capacity of 1262 mAh g^−1^ at 0.1 C. Under high sulfur loadings and/or elevated C-rates, CoWO_4_/WO_2_ substantially improves cycling stability relative to the single-phase counterparts. Overall, this study elucidates the catalytic mechanisms of CoWO_4_/WO_2_ heterostructures and offers a promising design strategy for high-performance Li–S battery catalysts.

## Experimental Section

### Synthesis of Catalysts

CoWO_4_/WO_2_ nanoparticles were synthesized using a hydrothermal method followed by hydrogen reduction. Two precursor solutions were prepared by dissolving Co(NO_3_)_2_·6H_2_O (291 mg) and Na_2_WO_4_·2H_2_O (330 mg) in deionized water, respectively. Add HCl to the solution of Na_2_WO_4_·2H_2_O to adjust the pH, then add the solution of Co(NO_3_)_2_·6H_2_O and mix it thoroughly. The two solutions were mixed under stirring for 20 min. The resulting mixture was then transferred to a Teflon-lined autoclave and heated at 180 °C for 2 h. After the reaction, the autoclave was allowed to cool naturally to room temperature. The resulting product is the precursor Co_4_W_6_O_21_(OH)_2_·4H_2_O. The obtained product was centrifuged and washed three times with deionized water to remove residual ions. The resulting solid was dried at 60 °C for 6 h and then annealed in a tube furnace under an Ar/H_2_ (95% Ar and 5% H_2_) atmosphere at 600 °C for 2 h to obtain the CoWO_4_/WO_2_ catalyst. Pristine CoWO_4_ was synthesized by mixing equimolar amounts of Co(NO_3_)_2_·6H_2_O and Na_2_WO_4_·2H_2_O to prepare the precursor solution. Similarly, WO_2_ was synthesized by mixing a 2:1 molar ratio of HCl and Na_2_WO_4_·2H_2_O in deionized water. After hydrothermal treatment, the procedures for washing, drying, and annealing followed the same steps as for the CoWO_4_/WO_2_ synthesis to obtain pure CoWO_4_ and WO_2_, respectively.

### Material Characterization

The crystal structure of the synthesized samples was analyzed using X-ray diffraction (XRD) within a Rigaku Smart Lab diffractometer. The morphology of the samples was characterized using scanning electron microscope (SEM, Zeiss Gemini 300) and transition electron microscope (TEM, JEM-F200). The chemical states of the elements were analyzed by X-ray photoelectron spectroscopy (XPS, Thermo Scientific K-Alpha). High-resolution TEM (HRTEM) simulation was performed with a commercial JEMS software.

### Visualization Tests of Polysulfide Adsorption

Li_2_S_4_ solution was prepared by mixing stoichiometric amounts of S and Li_2_S in a baseline electrolyte composed of 1.0 mol L^−1^ lithium bis-(trifluoromethanesulfonyl) imide and 2 wt% LiNO_3_ in a 1:1 volume ratio mixture of 1,2-dimethoxyethane (DME) and 1,3-dioxolane (DOL). CoWO_4_/WO_2_, CoWO_4_, and WO_2_ (50 mg) was added to the prepared Li_2_S_4_ solution (3 mL). After 5 h of adsorption, the supernatant was separated and diluted with DME/DOL. The diluted solution was analyzed by UV–visible spectroscopy (Agilent Cary5000). The remaining solid was filtered, dried, and analyzed by XPS. All experiments were conducted in an argon-filled glove box to avoid exposure to air.

### Symmetric Cell Tests

The catalysts, acetylene black (AB), and polyvinylidene fluoride (PVDF) were mixed in a weight ratio of 4:5:1 and dispersed in N-methylpyrrolidone (NMP) solvent. The resulting slurry was coated onto aluminum foil to form electrodes. The symmetric cell electrolyte was prepared by adding Li_2_S_4_ (20 mg mL^−1^) to the baseline electrolyte. Two identical electrodes were assembled with a Celgard 2300 separator in coin cells. Cyclic voltammetric (CV) tests were performed using a Biologic potentiostat (VSP, France) between − 0.8 and 0.8 V.

### Nucleation Experiments

The S-free cathode and Li foil were used as the cathode and anode, respectively. The Li_2_S_8_ electrolyte (20 μL) was precisely dispensed onto the cathode, while a 20 μL volume of the blank electrolyte was carefully introduced onto the anode. These two identical electrodes were assembled into coin cells. Initially, the resultant Li − S cells were galvanostatically discharged to 2.09 V and then polarized to 2.08 V. The current-time curves were immediately monitored after the voltage jump.

### Electrochemical Characterizations

S was loaded via a melt diffusion technique. Catalysts and S were first mixed in a sealed container and annealed at 155 °C for 6 h to facilitate S infiltration. The catalyst-sulfur mixture, AB, and PVDF were combined in a weight ratio of 7:2:1 and dispersed in NMP solvent. After stirring for 1 h, the slurry was coated onto aluminum foil using a doctor blade and vacuum-dried at 60 °C for 12 h. The S loading was controlled at 1.0 or 5 mg cm⁻^2^. Li − S coin cells were assembled with S-loaded cathodes, Celgard 2300 separators, and Li foil anodes. A 40 μL electrolyte was added to the cells. Galvanostatic charge-discharge tests were performed using a LAND testing system (Wuhan, China) in the voltage range from 1.7 to 2.6 V.

### Theoretical Calculations

The density functional theory (DFT) calculations were performed using the CASTEP program. The exchange–correlation interactions were treated with the Perdew–Burke–Ernzerhof functional under the generalized gradient approximation framework. The vacuum space was set to be 15 Å in the z direction, which was enough to negligible interactions between periodic units. During the construction of heterostructures, the lattice mismatch between constituent crystal phases was maintained below 5% to ensure structural stability at the heterointerface. The top surface atoms of each slab were allowed to relax, while the remaining atoms were constrained. The plane-wave cutoff energy was specified as 500 eV, and the Brillouin zone integration was performed using a 3 × 3 × 1 Monkhorst–Pack k-point mesh. The self-consistent field calculations employed convergence thresholds of 1 × 10⁻^5^ eV for total energy and 0.02 eV Å^−1^ for maximum atomic force. The adsorption energy (E_ads_) at the catalyst–polysulfide interface was evaluated through the relationship:

1 $${E}_{ads}={E}_{sub}+{E}_{polysulfide}-{E}_{total}$$

where ${E}_{total}$, ${E}_{sub}$, and ${E}_{polysulfide}$ denote the total energy of the adsorption system, the energy of the isolated catalyst substrate, and the energy of the free polysulfide species, respectively.

## Results and Discussion

### Autogenous Transformation for Heterostructured Catalysts

**Fig. 2:**
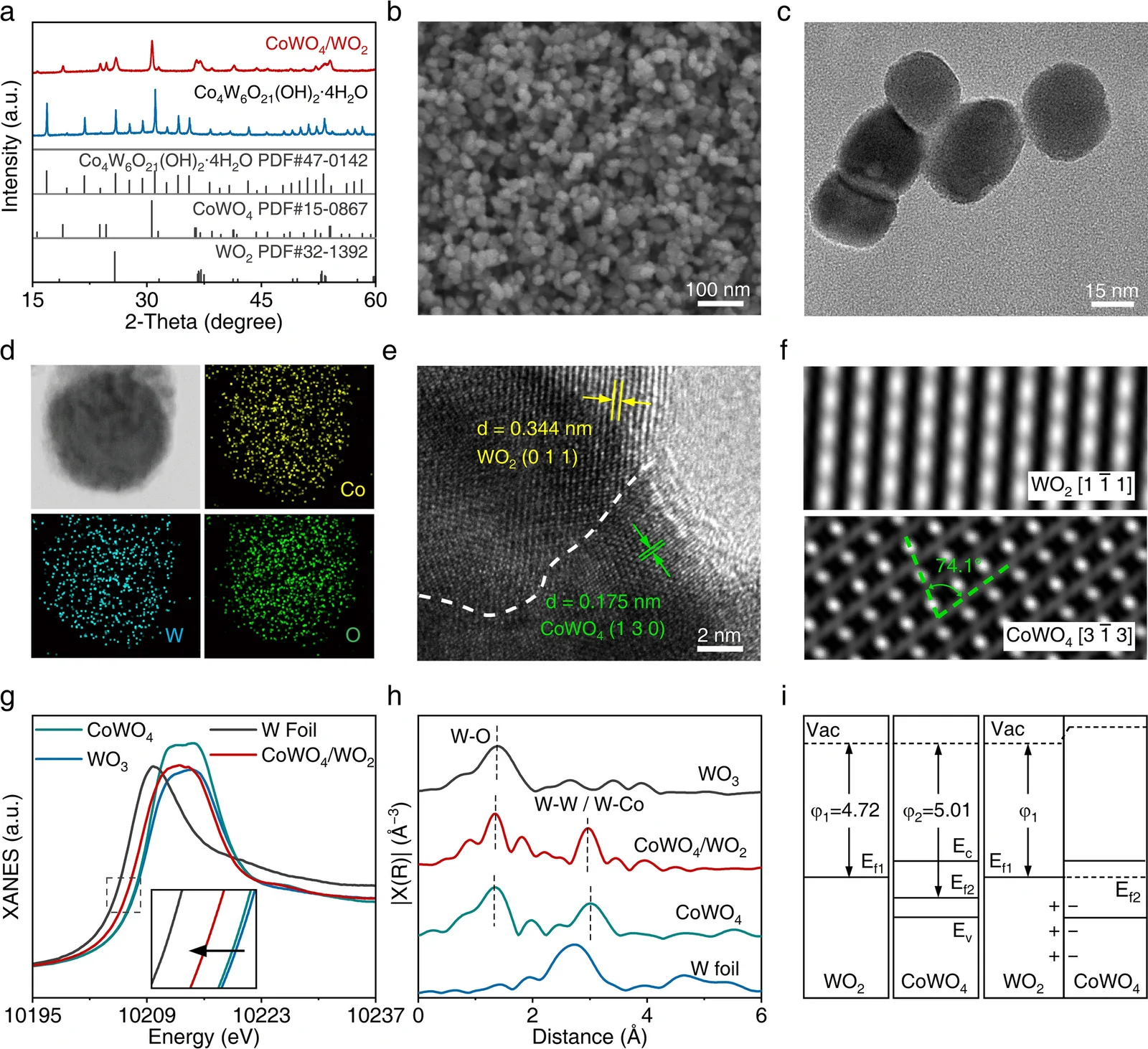
Material characterization of CoWO_4_/WO_2_ catalyst. **a** XRD patterns of precursor and heterostructures. **b** SEM images of CoWO_4_/WO_2_. **c** TEM images and **d** EDX spectrum of CoWO_4_/WO_2_ heterojunctions. **e** HRTEM and **f** Simulated images. **g** XANES and **h** FT of *k*^2^-weight χ(k) function for W L_3_ edges for W foil, WO_3_, CoWO_4_, and CoWO_4_/WO_2_. **i** Illustration of work functions difference between CoWO_4_ and WO_2_, and the electron transfer after their contact

The successful fabrication of CoWO_4_/WO_2_ heterostructures relies on precisely controlled precursor synthesis and subsequent treatment processes. Figure S1 presents the SEM image of the precursors synthesized via the hydrothermal method, revealing uniformly sized nanospheres. The corresponding XRD of the as-synthesized precursors (Fig. 2a) reveals well-defined diffraction peaks, which can be indexed to Co_4_W_6_O_21_(OH)_2_·4H_2_O [42]. Subsequent thermal annealing in a forming gas mixture (5% H_2_/95% Ar) induces autogenous phase transformation, as demonstrated by the emergence of distinct diffraction features corresponding to WO_2_ and CoWO_4_ (space group P2/*a*, PDF#15–0867) in the XRD pattern of post-annealed samples (Fig. 2a) [43]. The inductively coupled plasma optical emission spectroscopy (ICP-OES) analysis confirmed that the stoichiometric ratio of Co and W is close to 2:3 (Table S1). The SEM image of CoWO_4_/WO_2_ (Fig. 2b) has an average particle size of approximately 38 nm (also see the size distribution analysis in Fig. S2). The Brunauer–Emmett–Teller (BET) surface area was measured to be 28.67 m^2^ g^−1^ for CoWO_4_/WO_2_, while CoWO_4_ and WO_2_ show comparable values (Fig. S3). TEM analysis confirms successful crystallization of the nanoparticle ensemble (Fig. 2c). Energy-dispersive X-ray spectroscopy (EDX) elemental mapping (Fig. 2d) demonstrates homogeneous distribution of W, Co, and O across the nanospheres. However, the spatial resolution limitation of EDX precludes definitive observation of phase boundaries between WO_2_ and CoWO_4_ domains. HRTEM imaging (Fig. 2e) resolves lattice fringes corresponding to both the WO_2_ (011) plane (0.344 nm spacing) and the CoWO_4_ (130) (0.175 nm spacing) phases. Figure 2f presents the simulated HRTEM image generated using the commercial JEMS software. The observed lattice fringes were identified to the (011) plane of WO_2_. A $\left[1\overline{1 }1\right]$ zone axis was selected to be able to reproduce a similar lattice pattern. The simulated 2D atomic lattice image of CoWO_4_ exhibits well-matched interplanar spacings and angles between the ($20\overline{2 }$) and (130) planes, corresponding to the $\left[3\overline{1 }3\right]$ zone axis.

X-ray absorption spectroscopy (XAS) measurements were conducted on CoWO_4_/WO_2_ and compared with reference materials (CoWO_4_, WO_3_, and W foil). Figure 2g presents their W L_3_-edge X-ray absorption near-edge fine structure (XANES) [44, 45]. The W L_3_-edge energy of CoWO_4_ is around 10,207 eV, which is significantly higher than that of W foil and nearly overlapped with that of WO_3_, implying that the oxidation states of W may be approximately close to 6 + . After reduction with H_2_, the resultant CoWO_4_/WO_2_ heterojunction exhibits a lower adsorption edge than pristine CoWO_4_. The extended X-ray adsorption fine spectroscopy (EXAFS) of all four samples are presented in Fig. 2h. CoWO_4_/WO_2_ shows a broad peak at 1.34 Å (without phase correction), corresponding to the W − O scattering path. CoWO_4_ and WO_3_ have the major W − O peak at the similar position. The peak broadening observed in CoWO_4_ and WO_3_ originates from structural distortions in their WO_6_ octahedra, which leads to inequivalent WO bond lengths. The second peak of CoWO_4_/WO_2_ at 2.96 Å is attributed to the W − W and W − Co paths. In WO_2_, the two W − W paths arising from two dimmers of edge-sharing WO_6_ octahedra (Fig. S4) are shorter than the W − Co or W − W path in CoWO_4_, which causes the W − W/Co paths of CoWO_4_/WO_2_ (Fig. S5) to shift slightly toward a lower distance, consistent with the formation of heterojunctions. Complementary Co K-edge XANES analysis of CoWO_4_/WO_2_ (Fig. S6) demonstrates an intermediate oxidation state of Co between metallic foil and Co_3_O_4_ [46]. The appearance of significant pre-edge peaks is due to the distorted octahedral coordination of Co^2+^. The distinct pre-edge feature at 7710 eV arises from dipole-forbidden 1* s* → *3* *d* transitions characteristic of distorted [CoO_6_] environments.

To gain insights into the electronic structures of the heterojunction, the DFT was employed to elucidate the electronic properties of CoWO_4_ and WO_2_. Figure S7 presents the band structure and density of states (DOS) of pristine CoWO_4_, revealing it to be an indirect semiconductor with a band gap of 2.49 eV, which is consistent with the band gap width obtained from UV–vis measurements (Fig. S8) [47]. In contrast, WO_2_ displays metallic behavior with electron states crossing the Fermi level [48]. Work function calculations reveal a significant disparity between CoWO_4_ and WO_2_, which establishes an intrinsic electron transfer driving force across the heterointerface (Fig. S9) [49–51]. Kelvin probe force microscopy (KPFM) measurements further confirms this observation (Fig. S10). Upon contact, Fermi level equilibration induces spontaneous electron migration from WO_2_ to CoWO_4_, generating a built-in electric field at their interface (Fig. 2i). The interfacial charge transfer between WO_2_ and CoWO_4_ was further validated by Bader charge analysis (Fig. S11). Such a heterojunction holds advantages of good electron conduction and interfacial electric field, which are both favorable for sulfur conversion reactions.

### Catalytic Activities of Catalysis

**Fig. 3:**
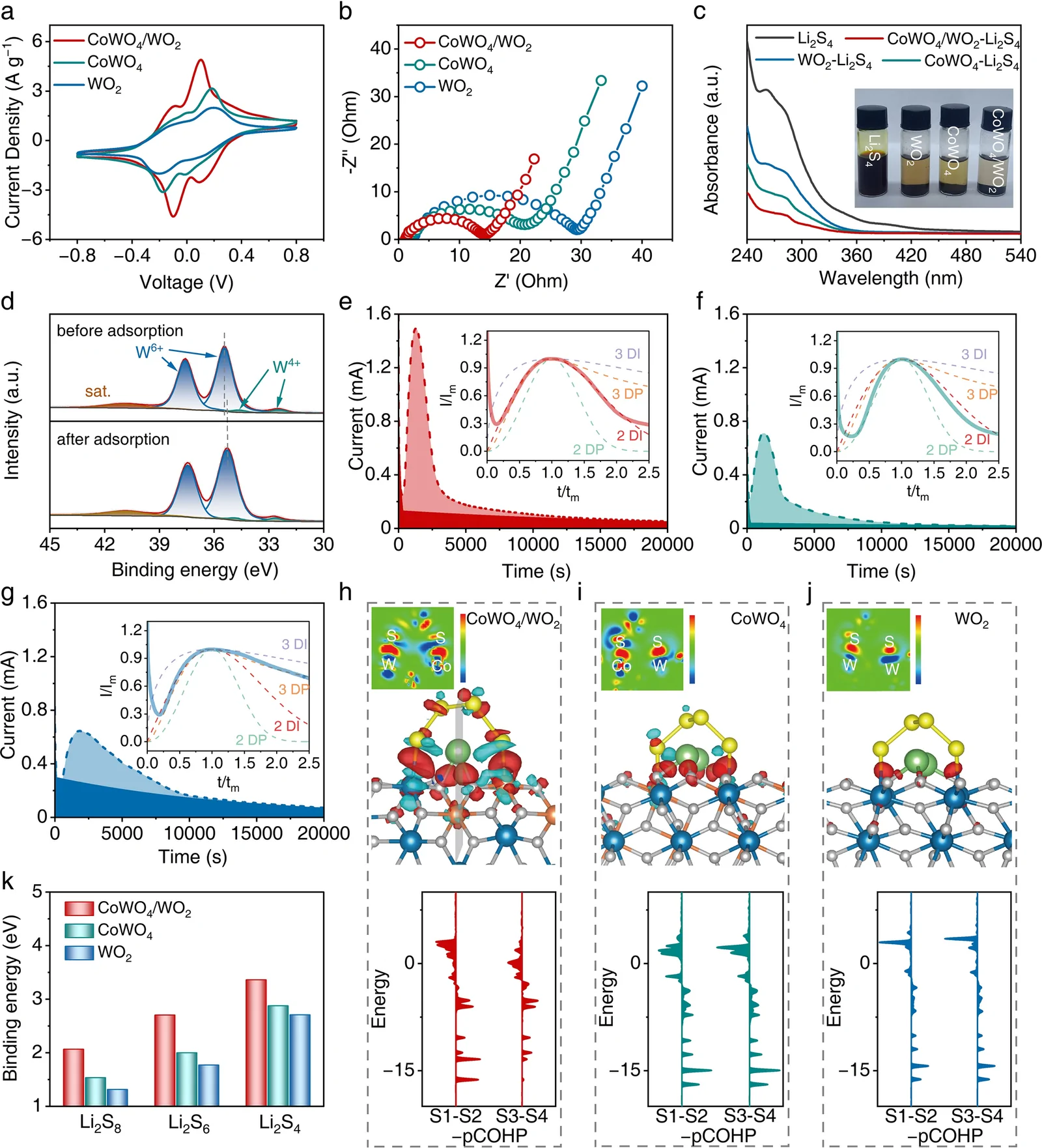
Catalytic properties and adsorption interaction between polysulfides and catalysts. **a** CV curves and **b** Nyquist plots of symmetric cells using different catalysts. **c** Visualization adsorption tests for CoWO_4_/WO_2_, CoWO_4_, and WO_2_. **d** XPS spectra of W 4*f* signal for CoWO_4_/WO_2_ before and after adsorption. Chronoamperometry curves of nucleation tests for different catalysts: **e** CoWO_4_/WO_2_, **f** CoWO_4_, and **g** WO_2_ (insets show the corresponding dimensionless transients compared with theoretical 2D and 3D models). The electron density difference diagrams of Li_2_S_4_ on **h** CoWO_4_/WO_2_, **i** CoWO_4_, and **j** WO_2_, along with the pCOHP curves of the S1 − S2 and S3 − S4 bonds activated by these catalysts. **k** Adsorption energy calculations of CoWO_4_/WO_2_, CoWO_4_, and WO_2_ for Li_2_S_8_, Li_2_S_6_, and Li_2_S_4_

To evaluate the catalytic performance, symmetric cells were first fabricated by uniformly loading equivalent catalyst masses (CoWO_4_/WO_2_ heterojunction, pristine CoWO_4_, and WO_2_) onto both cathodes and anodes using Li_2_S_4_-containing electrolyte for comparison. Figure 3a shows that the cyclic voltammetric (CV) curves of symmetric cells. Notably, the CoWO_4_/WO_2_ symmetric cell exhibited much higher redox peaks than the CoWO_4_ and WO_2_ cells. In addition, the redox peak voltages of the heterojunction cell are also closer to zeros. These CV characteristics imply that CoWO_4_/WO_2_ enhances the conversion kinetics, thereby demonstrating high current response and low overpotentials.

Furthermore, electrochemical impedance spectroscopy (EIS) was employed to measure the internal resistance of Li − S batteries using three catalysts. Nyquist plots (Fig. 3b) exhibit typical semicircles at high-frequency range and long linear tails at low- and medium-frequency range. These components can be modeled by an equivalent circuit consisting of a resistance and constant phase element (CPE) in parallel and Warburg impedance in series (Fig. S12). The resistance (*R*_ct_) of charge transfer for the CoWO_4_/WO_2_ cell was calculated to be 14.19 Ω, which is lower than those of either the CoWO_4_ (21.19 Ω) or WO_2_ (29.39 Ω) cells (Table S2). The enhanced kinetics of the CoWO_4_/WO_2_ heterojunction is correlated to the interaction between CoWO_4_/WO_2_ and polysulfides. Therefore, three catalysts were added to a Li_2_S_4_-containing electrolyte to monitor the Li_2_S_4_-catalyst interaction strengths by UV–Vis spectroscopy. After static adsorption for 5 h, the supernatant of CoWO_4_/WO_2_ exhibits almost transparent color whereas WO_2_ turns the brown electrolyte to light yellow (Fig. 3c), indicating that heterojunctions increase the adsorption strength of CoWO_4_/WO_2_ toward Li_2_S_4_. The intensity of the absorption band below 330 nm decreases as the following series: CoWO_4_/WO_2_ > CoWO_4_ > WO_2_, corroborating the interaction strength observed in the visualization tests.

The oxidation states of W, Co, and O were characterized by the XPS. The W 4*f* spectra (Fig. 3d) show two well-separated spin–orbit components at 35.45 and 37.62 eV, corresponding to W^6+^ in CoWO_4_, while the side peaks at 32.48 and 34.65 eV indicate the presence of W^4+^ in WO_2_. After adsorbing Li_2_S_4_, the W 4*f* peaks shift down to lower binding energies, indicating the electron transfer from polysulfide to catalysts [52]. The Co 2*p* signals in Fig. S13 shows broad peaks for both spin–orbit splitting components [53]. The typical satellite features indicate the existence of Co^2+^. Notably, after adsorbing Li_2_S_4_, an extra peak appears at the side of lower binding energy, implying that Co ions gain electrons from Li_2_S_4_ due to the strong Co − S interaction.

To reveal how the catalyst–polysulfide interaction influences the conversion reactions, we conducted Li_2_S_2_/Li_2_S nucleation experiments by galvanostatically discharging Li-S cells to 2.09 V and immediately polarizing them to 2.08 V to monitor the chronoamperometric curves [54]. The CoWO_4_/WO_2_ cell demonstrates a quick and sharp peak at 1285 s and delivers a high nucleation capacity of 349.74 mAh g^−1^ (Fig. 3e). A rapid nucleation results from the catalytic effect while the high capacity also confirms that catalysts convert more Li_2_S_2_/Li_2_S in a short time. The rapid kinetics of heterojunctions forms a stark contrast against pristine CoWO_4_ and WO_2_ (Fig. 3f, g). Furthermore, the current–time curves were fitted with the Scharifker–Hills models (Table S3). Figure 3e shows that CoWO_4_/WO_2_ induces the 2D instantaneous nucleation (2DI) owing to rapid catalytic reactions and deposition on the surface. In contrast, CoWO_4_ follows a combination of 2DI and 2D progressive nucleation (2DP) models, resulting in lower capacity and prolonged time, while WO_2_ exhibits the 3D progressive nucleation (3DP) behavior, which results from poor kinetics and liquid-phase disproportionation.

To further understand the polysulfide–catalysts interaction, we calculated the electron density difference diagrams of Li_2_S_4_ on three catalysts and sliced 2D images through key interacting atoms (Fig. 3h − j). Two terminal sulfur atoms were attracted to cations, forming the Co-S or W-S bonds because electrons typically accumulate along the connection line, indicative of covalent character. In addition, Li-ions were also attached to oxygen anions with more ionic interactions. The 2D slice images show that the CoWO_4_/WO_2_ heterojunctions induce more significant electron redistribution, signifying strong interaction as compared to CoWO_4_ and WO_2_. Adsorption is an energy downhill process, in which electrons in S−S covalent bonds are redistributed to the M-S bonds. In principle, the stronger M-S bonds are, more favorable the process is. In addition, the heterogeneous site pattern enables elongation of S-S bonds of Li_2_S_4_ (Fig. S14), indicating that the heterojunction interface strongly strengthens its interaction with polysulfides and enhances bond activation. To quantify this bond activation, we calculated the projected crystal orbital Hamilton population (pCOHP) for the S1 - S2 and S3 - S4 bonds of Li_2_S_4_ adsorbed on different catalysts. As shown in Fig. 3h − j, the integrated pCOHP (iCOHP) values of the terminal S1-S2 (− 4.92 eV) and S3−S4 (− 1.34 eV) bonds adsorbed on CoWO_4_/WO_2_ are less negative than those on CoWO_4_ (− 5.43 eV, − 5.26 eV) and WO_2_ (− 5.64 eV, − 5.33 eV), indicating that the S−S bonds on CoWO_4_/WO_2_ are weakened (or activated). Therefore, polysulfide destabilization mediated by the CoWO_4_/WO_2_ heterojunction leads to a lower dissociation barrier for subsequent conversion steps. Additionally, this synergistic electronic modulation enables uniform high-efficiency Li_2_S deposition and improved sulfur utilization. Figure 3k presents the calculated adsorption energies of three polysulfide molecules (Li_2_S_8_, Li_2_S_6_, and Li_2_S_4_) on three catalysts (see the adsorption models in Fig. S15). CoWO_4_/WO_2_ demonstrates the highest adsorption energy for these polysulfides, corroborating the observed visualization and activation phenomena. These experimental and theoretical analyses conclude that the CoWO_4_/WO_2_ heterojunction significantly enhances catalytic performance by improving the adsorption and activation capabilities of polysulfides.

### Electrochemical Performance of Li − S Full Cells

**Fig. 4:**
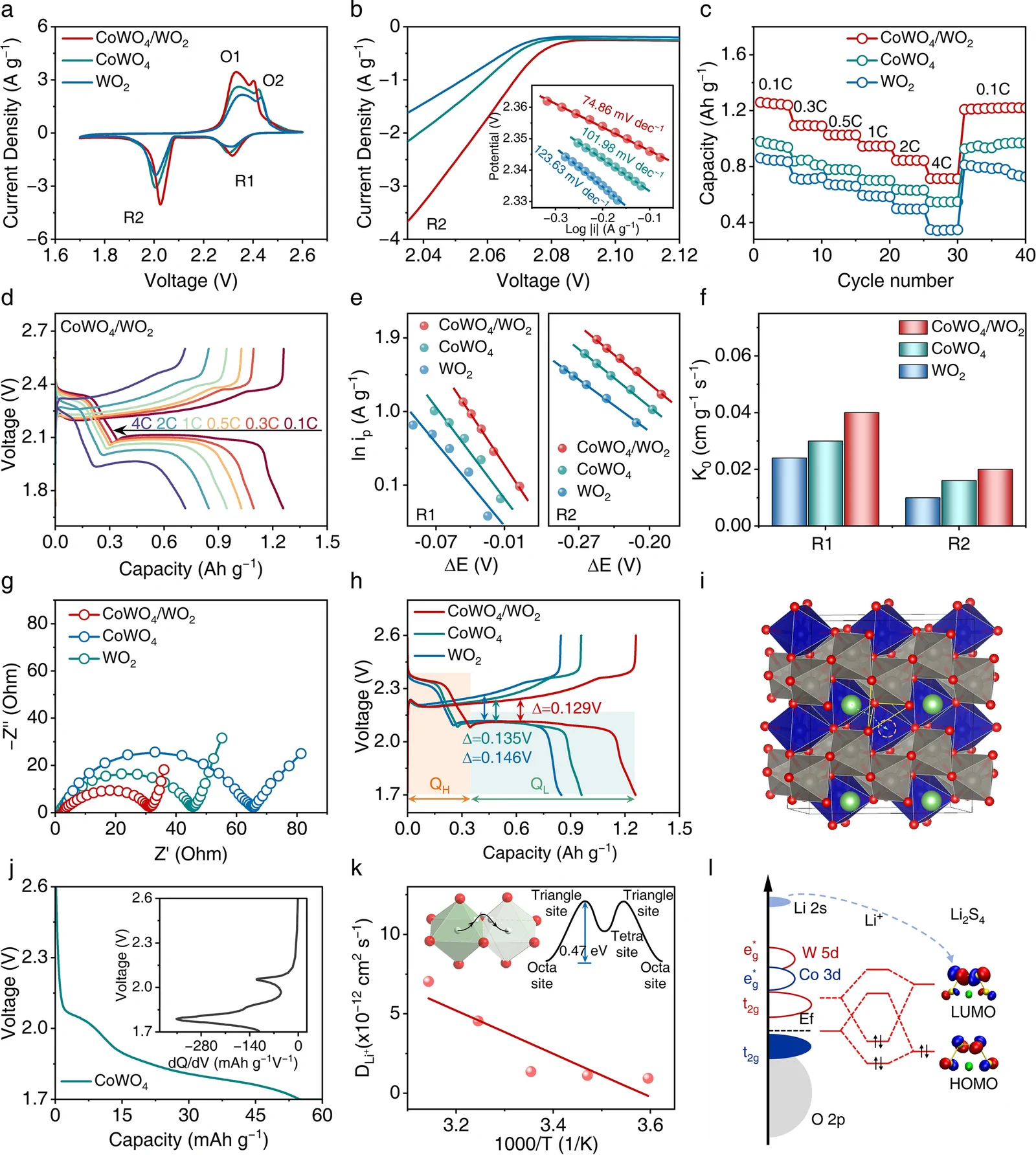
Electrocatalytic properties of Li–S batteries. **a** CV curves of CoWO_4_/WO_2_, CoWO_4_, and WO_2_. **b** Tafel slope of peak R2 in the CV curves. **c** Capacity retention at different rates. **d** Galvanostatic charge–discharge profiles of the CoWO_4_/WO_2_ Li-S batteries at different rates. **e** Relation between CV peak currents and Δ*E* for the CoWO_4_/WO_2_, CoWO_4_, and WO_2_ cells. **f** Rate constant ${k}_{0}$ values of the three catalysts. **g** Nyquist plots of Li − S batteries. **h** Constant current charge–discharge curves at 0.1 C. **i** Illustration of Li-ion diffusion in CoWO_4_. **j** Discharge curves and dQ/dV vs. capacity curves of CoWO_4_ Li-ion batteries. **k** Diffusion rate of CoWO_4_ Li-ion batteries varies with temperature. **l** Orbital interactions between polysulfides and catalysts

Figure 4a presents the CV curves of Li-S cells employing CoWO_4_/WO_2_, CoWO_4_, and WO_2_ as catalysts, recorded within the voltage range of 1.7-2.6 V at a scan rate of 0.1 mV s^−1^. All three cells exhibit two distinct reduction peaks (R1 and R2) and two partly overlapping oxidation peaks (O1 and O2). The reduction peaks (R1 and R2) correspond to the sequential transformation of solid S_8_ into long-chain polysulfides (Li_2_S_x_, x ≥ 4), followed by their further reduction to insoluble Li_2_S_2_/Li_2_S. The oxidation peaks (O1 and O2) reflect the gradual re-oxidation of Li_2_S back to S_8_. The clear separation of these peaks imply rapid kinetics relative to liquid disproportionation reactions. Notably, the CoWO_4_/WO_2_-based cell exhibits the highest R2 peak intensity, with its potential closer to the thermodynamic equilibrium compared to cells with CoWO_4_ or WO_2_ alone. These characteristics indicate that CoWO_4_/WO_2_ effectively accelerates polysulfide conversion in Li − S cells.

To further assess the electrocatalytic kinetics, the exponential region of the CV curves (between the linear and diffusion-limited ranges) was extracted and fitted to the Tafel model (Figs. 4b and S16). As depicted in Fig. 4b, the CoWO_4_/WO_2_ heterojunction yields a significantly lower Tafel slope (74.86 mV dec^−1^) compared to pristine CoWO_4_ (101.98 mV dec^−1^) and WO_2_ (123.63 mV dec^−1^). This result suggests that CoWO_4_/WO_2_ requires an overpotential of merely ~ 75 mV to achieve a tenfold increase in current density, underscoring its superior catalytic activity. Consequently, the CoWO_4_/WO_2_-based cell exhibits reduced polarization losses, enabling greater energy release during discharge and lower energy consumption during recharge, which ultimately enhances its rate performance.

Figure 4c presents the rate capability of the three Li–S cells at varying C-rates. The CoWO_4_/WO_2_-based cell achieves a high specific capacity of 1259 mAh g^−1^ at 0.1 C, which decreases progressively with increasing current rates but recovers to 1204 mAh g^−1^ upon returning to 0.1 C. In contrast, both CoWO_4_ and WO_2_ exhibit lower specific capacities at 0.1 C and undergo more pronounced capacity degradation after cycling at high rates. Figures 4d and S17 present the charge/discharge curves of CoWO_4_/WO_2_, CoWO_4_ and WO_2_ at different rates. Clearly, the discharge capacity of Li − S batteries using CoWO_4_/WO_2_ is higher than those cells using CoWO_4_ and WO_2_. The CoWO_4_/WO_2_ battery exhibits longer discharge plateaus during cycling and consistently maintains two complete discharge plateaus, even at a high current density of 4 C. In contrast, the discharge plateaus of the CoWO_4_ and WO_2_ batteries become incomplete as the current density increases, implying poor kinetics.

To further analyze the reaction kinetics, CV measurements were performed at various scan rates (Fig. S18). The peak current density (${i}_{p}$) is correlated with the formal potential (${E}^{0{\prime}}$), the peak voltage (${E}_{p}$), and the rate constant (${k}_{0}$), which was fitted using the following equation [55] under the assumption of an irreversible process:

2 $${i}_{p}=0.227FA{C}_{0}{k}_{0}\mathrm{exp}[-\alpha F({E}_{p}-{E}^{0{\prime}})/(RT)]$$

where F is the Faraday constant, A is the surface area, ${C}_{0}$ is the concentration of the reactants, α is the transfer coefficient, R is the gas constant, and T is the temperature. Figure 4e displays the extracted data and the corresponding fitting curves of ln (${i}_{p}$) vs. Δ*E* (${E}_{p}-{E}^{0{\prime}}$). The derived rate constants (Fig. 4f) at the R1 and R2 peaks are 0.041 cm g^−1^ s^−1^ and 0.020 cm g^−1^ s^−1^, respectively, for CoWO_4_/WO_2_, which are significantly higher than those for CoWO_4_ and WO_2_. These results confirm the intrinsically superior catalytic activity of the CoWO_4_/WO_2_ heterojunction. The rapid charge transfer kinetics are further validated by an EIS analysis (Fig. 4g, Table S4), where the CoWO_4_/WO_2_ cell exhibits a markedly reduced semicircle, indicative of a lower charge transfer resistance.

The galvanostatic charge–discharge profiles at 0.1 C are shown in Fig. 4h. The CoWO_4_/WO_2_-based cell delivers a high specific capacity of 1262 mAh g^−1^, surpassing those of the CoWO_4_ and WO_2_ cells under identical conditions. The discharge profiles feature two distinct voltage plateaus, corresponding to the previously observed reduction peaks in CV. Theoretically, the capacity ratio between the low-voltage (Q_L_) and high-voltage (Q_H_) plateaus is expected to be 3:1 (Fig. S19). The CoWO_4_/WO_2_-based cell exhibits a higher Q_L_/Q_H_ ratio than the CoWO_4_ and WO_2_ cells, suggesting that the latter materials exhibit insufficient catalytic activity, particularly at the second reduction plateau. Additionally, the half-capacity voltage gap (Δ*E*_hcvg_), a critical metric for evaluating reaction kinetics, was determined. The CoWO_4_/WO_2_ cell exhibits a low Δ*E*_hcvg_ of only 0.129 V, indicative of minimized polarization due to enhanced catalytic kinetics. These CV, EIS, and kinetic analyses reinforce the conclusion that the CoWO_4_/WO_2_ heterojunction effectively promotes polysulfide conversion reactions in Li–S batteries.

Although the atomic-level simulations (Fig. 3h − j) provide thermodynamic insights into the superior catalytic performance of CoWO_4_/WO_2_, whether additional mechanisms exist remains an open question. A detailed examination of the crystallographic structure of CoWO_4_ may offer further understanding of its thermodynamic activation, kinetic enhancement, and ion transport behavior. In CoWO_4_, both Co and W atoms are coordinated with six oxygen atoms, forming [CoO_6_] and [WO_6_] octahedral units (Fig. 4i). Along the *x*-direction, CoWO_4_ exhibits a layered structure with alternative alignment of [CoO_6_] or [WO_6_] octahedral layers. Each layer is half filled with zig-zag chains of edge-sharing [CoO_6_] or [WO_6_] octahedra. Between adjacent layers, [CoO_6_] octahedra from one layer share corners with [WO_6_] octahedra from the neighboring layer. Such a unique structure opens a directional channel of vacant octahedral channel along the c-axis, which may serve as a fast Li-ion diffusion pathway.

To validate this hypothesis, CoWO_4_ was employed as a cathode material in an assembled Li-ion battery in the absence of sulfur species. The discharge curves within the voltage range of Li–S cells (Fig. 4j) indicate that CoWO_4_ delivers a moderate specific capacity of approximately 53 mAh g^−1^, implying that Li–ions intercalate into the vacant octahedral sites. More importantly, the discharge plateaus closely resemble those of Li–S batteries, indicating that, during Li–S cell operation, CoWO_4_ undergoes partial lithiation. This suggests that CoWO_4_ may function as a Li-ion reservoir or a transport medium. To further corroborate this assumption, the Li–ion migration energy barrier along the diffusion channel was computed. Based on a classical hopping model, a Li ion transitions from one octahedral site through a triangular bottleneck to an intermediate tetrahedral site, before reaching the final octahedral site. DFT calculations indicate that the energy barrier at the bottleneck is only 0.47 eV. Moreover, experimental measurements of diffusion coefficients at various temperatures (Fig. 4k) yield a room-temperature Li-ion diffusivity of 1.38 × 10^−12^ cm^2^ s^−1^. The activation energy for Li-ion diffusion, determined through Arrhenius fitting, is calculated to be 41.57 kJ mol^−1^, confirming the rapid diffusion of Li–ions.

Based on the above analyses, a catalytic mechanism of the CoWO_4_/WO_2_ heterojunction is proposed, as illustrated in Fig. 4l. CoWO_4_ exhibits a strong adsorption capability, effectively immobilizing polysulfides intermediates, however, its inherently poor electronic conductivity limits charge transfer. In contrast, the in situ-formed WO_2_ provides an efficient electron transport pathway. Additionally, the CoWO_4_/WO_2_ heterojunction weakens S − S bonds and activates polysulfides by lowering the energy barrier for bond breaking. Under negative polarization, electrons migrate to the CoWO_4_/WO_2_ interface and are injected primarily into the 5*d* orbitals of W cations (with minimal transfer to Co 3*d* orbitals). At the heterojunction interface, asymmetric electronic interactions occur between the catalyst and the adsorbed polysulfides. Excess electrons subsequently populate the antibonding orbitals of the S–S backbone, thereby forming nucleophilic centers. Simultaneously, the partial lithiation of CoWO_4_ facilitates Li-ion transport along its intrinsic diffusion channels, enabling the formation of S–Li bonds at the reaction sites. With the combined effects of electron injection and Li-ion transport, the S–S bonds weakened by catalyst–polysulfide interactions consequently dissociate into short-chain polysulfides. Thus, the CoWO_4_/WO_2_ heterojunction synergistically integrates multiple functionalities, including strong polysulfide adsorption, asymmetric orbital interactions, bond weakening, and efficient ion–electron transport pathways.

### In-situ Characterization of Li − S Full Cells

**Fig. 5:**
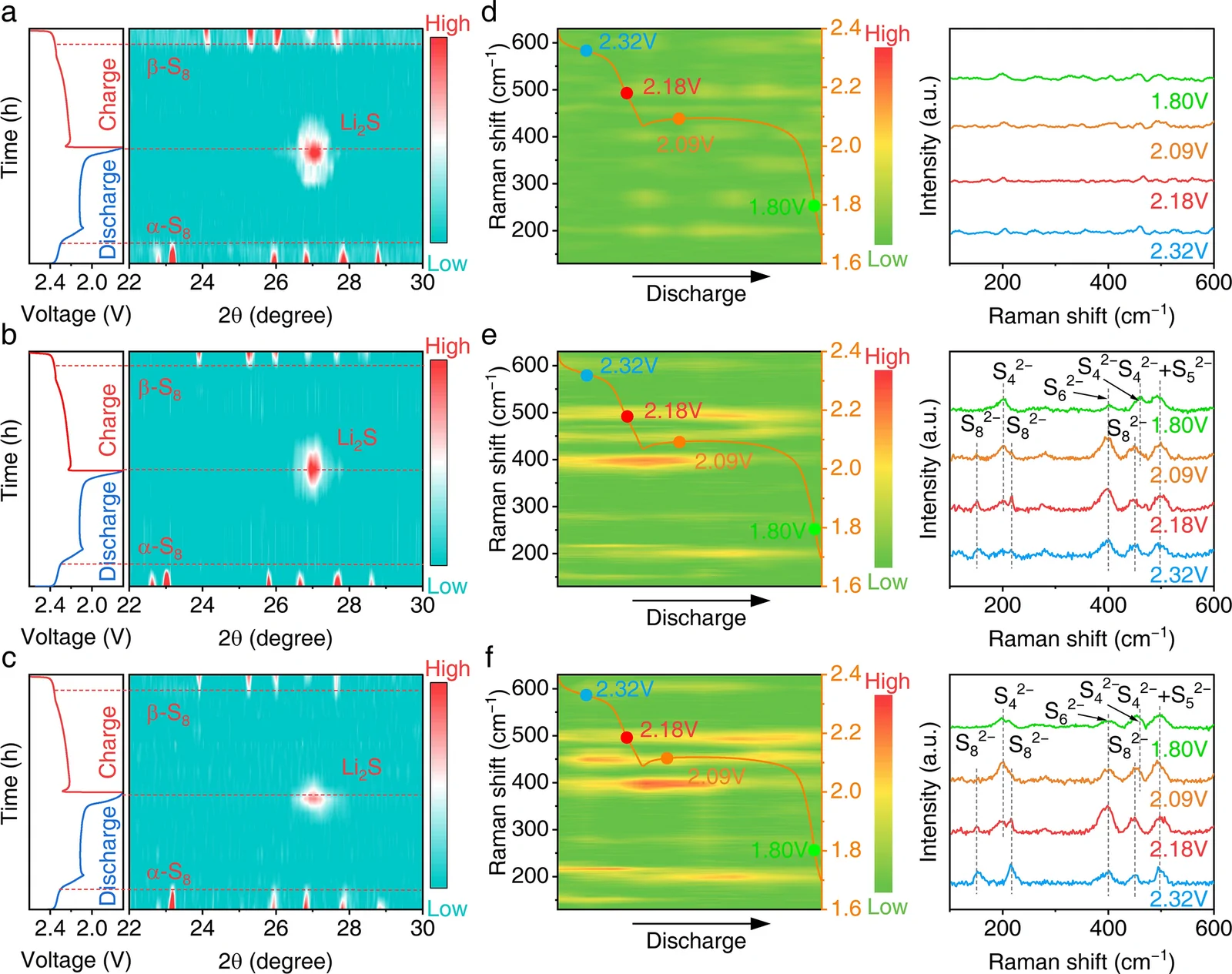
In-situ characterization of Li–S batteries. **a-c** In-situ XRD testing and **d-f** in-situ Raman testing of CoWO_4_/WO_2_, CoWO_4_, and WO_2_

Figure 5a presents the in-situ XRD results of sulfur species that were catalyzed and converted during cycling. At the beginning of discharge, a distinct ɑ-S_8_ peak is observed in the cathode. As discharge progresses, this peak gradually disappears toward the end of the high-voltage plateau, indicating a rapid transition of sulfur to amorphous states. During the second half of the low-voltage plateau, a broad peak centered at 2θ = 26.9° emerges and gradually increases in intensity until it reaches its maximum at 1.7 V. This broad peak is attributed to poorly crystalline Li_2_S. Upon charging, the intensity of this Li_2_S peak diminishes gradually and vanishes completely by the end of the charge cycle. As charging nears completion, a crystalline sulfur peak (β-S_8_) appears and becomes most pronounced at the end of the charge [56–59]. Figure 5b, c presents the in-situ XRD results for CoWO_4_ and WO_2_, respectively. Compared to CoWO_4_ and WO_2_, the CoWO_4_/WO_2_ electrode exhibits the strongest β-S_8_ peak from the formed Li_2_S. These results highlight that the CoWO_4_/WO_2_ heterojunction enhances the efficiency of polysulfide conversion during cycling, thus accelerating the reaction kinetics.

To further investigate the suppression of the polysulfide shuttle effect by CoWO_4_/WO_2_, in-situ Raman spectroscopy was employed to monitor changes at the anode during discharge. Figure S20 illustrates a schematic of the in-situ Raman setup, where the laser detects polysulfide on the anode side through a quartz window. Figure 5d shows that no distinct polysulfide Raman peaks are observed on the anode during discharge for CoWO_4_/WO_2_. However, Fig. 5e displays clear polysulfide peaks in the Raman spectra for CoWO_4_ (highlighted in orange). At 2.32 V, several Raman peaks in the initial discharge stage correspond to S_8_^2−^ (150, 219, and 454 cm^−1^), along with minor peaks from S_6_^2−^ (400 cm^−1^)、S_4_^2−^ (498 cm^−1^) and S_5_^2−^ (500 cm^−1^) [60, 61]. As the reaction proceeds, the intensity of the S_8_^2−^ peaks gradually decreases but does not completely vanish. The peaks for S_6_^2−^ (400 and 506 cm^−1^), S_4_^2−^ (203, 460, and 498 cm^−1^), and S_5_^2−^ (500 cm^−1^) gradually increase and persist throughout the discharge cycle [62, 63]. The Raman spectrum for the WO_2_ cell anode shows slightly stronger polysulfide peaks compared to CoWO_4_ (Fig. 5f). The results confirm substantial polysulfide shuttling on CoWO_4_ and WO_2_ electrodes, along with slow conversion kinetics from S_8_ to Li_2_S, contributing to capacity loss in the battery. In contrast, the CoWO_4_/WO_2_ heterojunction effectively mitigates the shuttle effect, enhancing the sulfur utilization.

### Battery Cycling and Post-mortem Analysis

**Fig. 6:**
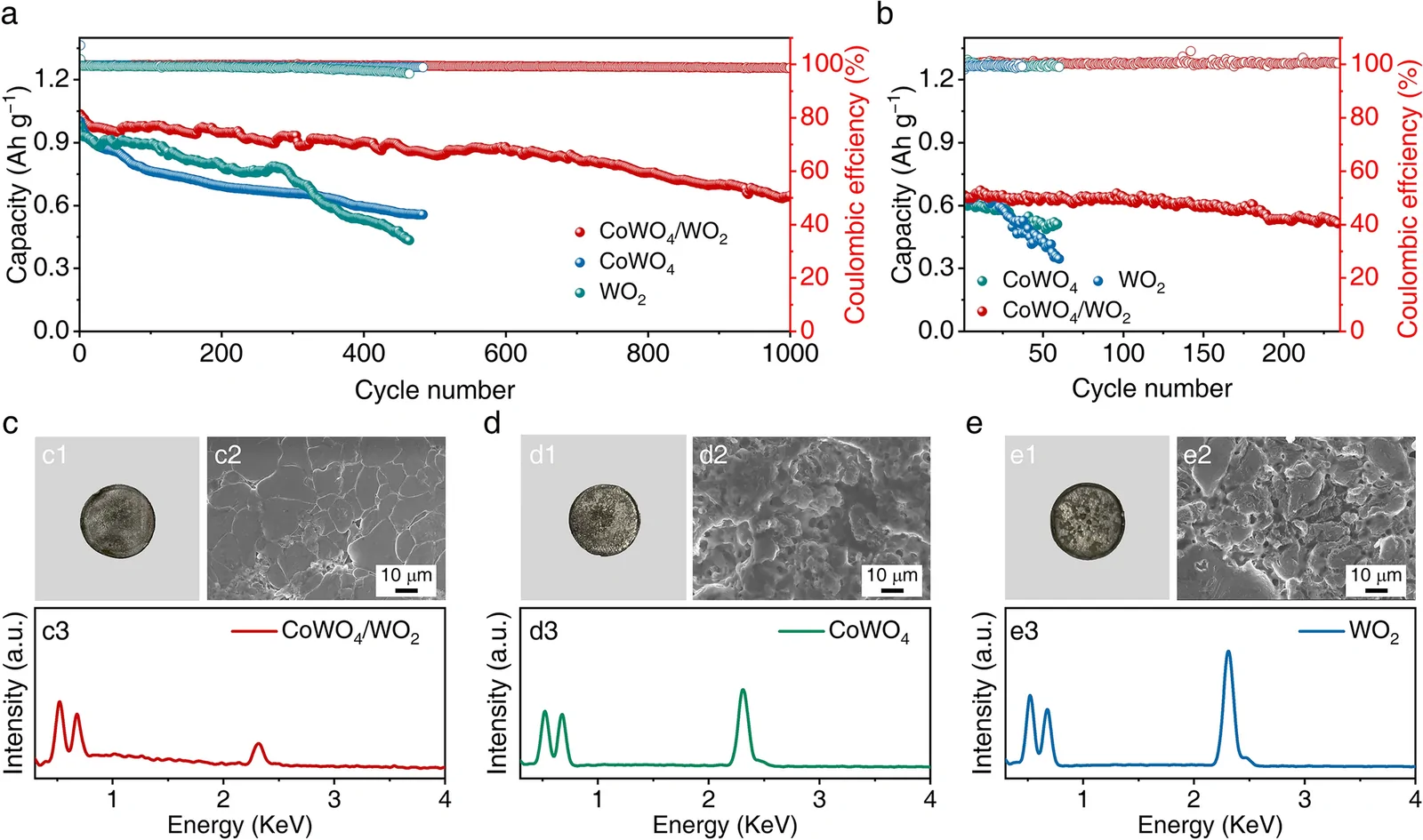
Battery cycling and post-mortem analysis. **a** Long-term cycling performance of the CoWO_4_/WO_2_, CoWO_4_, and WO_2_ cells at 1 C. **b** Capacity retention at high areal S loading (5 mg cm^−2^). Optical photographs, SEM images, and EDX plots of the disassembled anodes of **c** CoWO_4_/WO_2_, **d** CoWO_4_, and **e** WO_2_ cells after long-term cycling

To study the impact of catalysts on the long-term cycling stability of Li − S batteries, galvanostatic cycling tests were performed with a S loading of 1 mg cm^−2^ at 1 C, as shown in Fig. 6a. The CoWO_4_/WO_2_ cell delivers an initial capacity of 1035.7 mAh g^−1^ and retains 62.4% of its initial capacity after 1000 cycles, demonstrating a low decay rate of 0.038% per cycle. The CE remains as high as 98.7%. The cycling properties are superior to the previous reports of Co or W oxides as the catalysts as shown in Table S5). Figure S21 presents the charge/discharge curves of CoWO_4_/WO_2_ during a long-cycle test. The charge–discharge curve maintains a well-defined voltage plateau, and the capacity gradually decreases over time. This result demonstrates the good long-cycle stability of the CoWO_4_/WO_2_ electrodes. In contrast, due to its poor electronic conductivity and catalytic activity, the CoWO_4_ cell maintains only 55.6% of its initial capacity after 483 cycles. The WO_2_ cell exhibited a rapid capacity decay, dropping to 434.7 mAh g^−1^, after 283 cycles, likely resulting from the limited catalytic activity. Figure 6b also shows the cycling performance of the three cells with a high sulfur loading of 5 mg cm⁻^2^. After 60 cycles, CoWO_4_ retained only 84.6% of its initial capacity, while WO_2_ retained 53.2%. In comparison, the CoWO_4_/WO_2_ electrode maintained 79.1% of its initial capacity after 235 cycles. These results from both high-loading and long-cycle tests demonstrate that the CoWO_4_/WO_2_ heterojunction can significantly enhances the stability of Li − S batteries due to its superior catalytic effects.

To further understand the shuttling effects, post-mortem analysis was conducted on the disassembled anodes after 200 cycles. Figure 6c − e shows the optical (c1 − e1) and SEM images (c2 − e2) of the cycled Li foil. The metallic Li anode in the cycled CoWO_4_ and WO_2_ cells displays rough surfaces and have more yellow deposits. The EDX analysis reveals relatively high sulfur signals, indicating substantial polysulfide migration from the cathode to the anode. Conversely, the Li anode in the CoWO_4_/WO_2_ cell displays a relatively smooth surface with lower sulfur signals in EDX, suggesting improved catalytic effects and inhibition of polysulfide migration. These results demonstrate that the CoWO_4_/WO_2_ heterojunction not only achieves the effective anchoring of polysulfides through strong chemical adsorption, but also promotes the rapid transformation kinetics of polysulfides, thereby inhibiting the shuttle effect.

## Conclusions

In this study, we designed a heterojunction (CoWO_4_/WO_2_) for to catalyzed Li − S conversion reactions. Through a hydrothermal synthesis followed by a H_2_ reduction, the nanoscale heterojunction of CoWO_4_/WO_2_ was fabricated successfully. Such an architecture includes the following design strategies: (1) the strong adsorption of CoWO_4_ can adsorb polysulfides to suppress shuttling; (2) the heterogeneous interface can activate polysulfides and lower the reaction barrier; (3) metallic WO_2_ offers good electron conductivity; (4) direction channels of CoWO_4_ provides rapid Li-ion pathway and serves as a Li-ion reservoir; Benefiting from the synergy of multifunctionalities, the CoWO_4_/WO_2_ heterojunction dramatically accelerates polysulfide conversion and suppresses the shuttling effects, thereby demonstrating superior electrochemical properties. The Li − S batteries CoWO_4_/WO_2_ can deliver a high capacity of 1262 mAh g^−1^ at 0.1 C and exhibit a minimal capacity decay (0.038% per cycle) after 1000 cycles at 1 mg cm⁻^2^ S loading. At 5 mg cm⁻^2^ loading, 79.1% of its initial capacity can be retained after 235 cycles. This work reports a promising strategy for improving the cycling performance and efficiency of Li − S batteries and provides an alternative approach to designing high-efficiency catalysts.

## Supplementary Information

Below is the link to the electronic supplementary material.

Supplementary file1 (DOCX 11259 KB)
