# Supplementary material for: Metallic WO2-Promoted CoWO4/WO2 Heterojunction with Intercalation-Mediated Catalysis for Lithium–Sulfur Batteries
Source: Nanomicro Lett. 2025 Jul 18;18:7. doi: 10.1007/s40820-025-01849-3 (PMC12274174; doi:10.1007/s40820-025-01849-3)
Supplement: Supplementary file 1 — Supplementary file1 (DOCX 11259 KB) [file 40820_2025_1849_MOESM1_ESM.docx]

Supporting Information for

**Metallic WO_2_-Promoted CoWO_4_/WO_2_ Heterojunction with Intercalation-Mediated Catalysis for Lithium–Sulfur Batteries**

Chan Wang^1, 3^, Pengfei Zhang^2^, Jiatong Li^3^, Rui Wang^3^, Changheng Yang^3^, Fushuai Yu^2^, Xuening Zhao^3^, Kaichen Zhao^2^, Xiaoyan Zheng^2,^ *, Huigang Zhang^2, 3, 4,^ *, Tao Yang^1,^ *

^1^ Shaanxi Key Laboratory for Theoretical Physics Frontiers, Institute of Modern Physics, Northwest University, Xi’an 710127, P. R. China

^2^ Shaanxi Key Laboratory of Degradable Biomedical Materials, School of Chemical and Engineering, Institute of Low-Carbon Technology Application, Northwest University, Xi’an, Shaanxi 710069, P. R. China

^3^ State Key Laboratory of Mesoscience and Engineering, Institute of Process Engineering, Chinese Academy of Sciences, Beijing 100190, P. R. China

^4^ School of Chemical Engineering, University of the Chinese Academy of Sciences, No. 19(A) Yuquan Road, Shijingshan District, Beijing 100049, P. R. China

*Corresponding authors. E-mail: [zy129@nwu.edu.cn](mailto:zy129@nwu.edu.cn) (Xiaoyan Zheng); [hgzhang@ipe.ac.cn](mailto:hgzhang@ipe.ac.cn) (Huigang Zhang); [yangt@nwu.edu.cn](mailto:yangt@nwu.edu.cn) (Tao Yang)

**S1 Supplementary Methods**

**S1.1 In-situ XRD Diffraction**

The crystal structure evolution of sulfur species was investigated using in situ XRD diffraction (Rigaku Smart Lab) under operando electrochemical conditions. Assemble the in-situ electrochemical cell in the order of sulfur cathode/electrolyte/separator/lithium foil anode. During galvanostatic cycling at 0.2 C, XRD patterns were collected in real time through a beryllium window using Cu K$\alpha$ radiation (*λ* = 1.5406 Å), with a scan range of 20°-40° (2*θ*) and a step size of 10°. Phase transitions of sulfur species, including elemental sulfur (S_8_), lithium polysulfides (Li_2_S_x_), and lithium sulfide (Li_2_S), were analyzed based on characteristic diffraction peaks and their dynamic evolution.

**S1.2 In-situ Raman Spectroscopy**

The chemical evolution of LiPSs was monitored using a Lab RAM Odyssey Raman spectrometer (HORIBA FRANCE SAS). An in situ Raman cell, equipped with a quartz glass window for laser transmission and signal collection, was assembled as follows: cathode shell, lithium foil with an aperture (to enable optical access), separator, anode, and anode shell. During galvanostatic discharge at a 0.2 C rate, Raman spectra were collected through the quartz window using 532 nm laser excitation, with a spectral range of 100-600 cm^−1^ to capture key vibrational modes of sulfur species. The aperture in the lithium foil facilitated unobstructed laser penetration for real-time detection of LiPSs characteristic bands.

**S1.3 XAS Measurements**

Transmission XAS measurements were performed on a laboratory device of easyXAFS300+ (easyXAFS LLC). XANES and EXAFS of catalyst Co K-edge and W L3-edge were collected under the same conditions, and calibrated using metal Co foil and W foil. Data analysis was performed using the Athena software in order to normalize the spectrum.

**S1.4 Kelvin Probe Force Microscopy Measurements**

The measurements were performed using a Bruker Dimension Icon atomic force microscope (AFM) from Germany. First, the dispersed sample was drop-cast onto a conductive substrate and allowed to dry naturally. A SCM-PIT-V2 probe was then selected to conduct characterization in Kelvin probe force microscopy (KPFM) mode. After testing, the work function of the catalyst was calculated and calibrated using a gold (Au) reference standard.

**S2 Supplementary Figures and Tables**


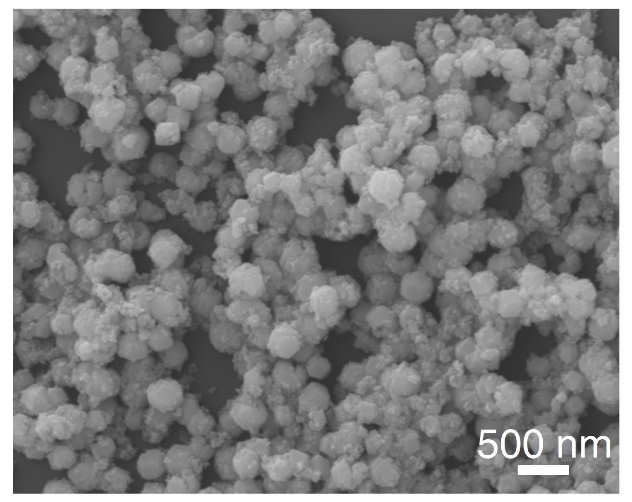


**Fig. S1** SEM images of Co_4_W_6_O_21_(OH)_2_·4H_2_O


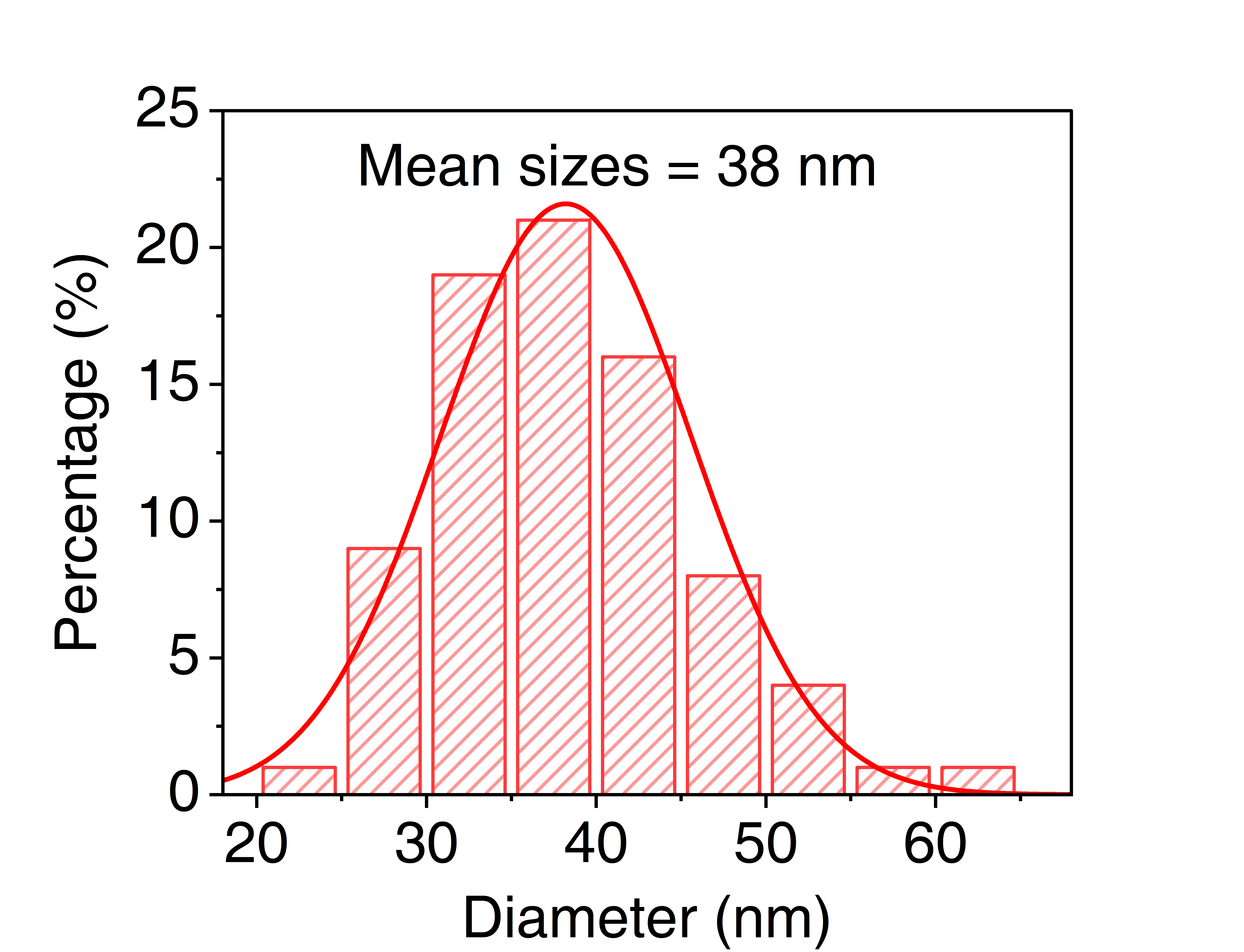


**Fig. S2** Particle size distribution of CoWO_4_/WO_2_


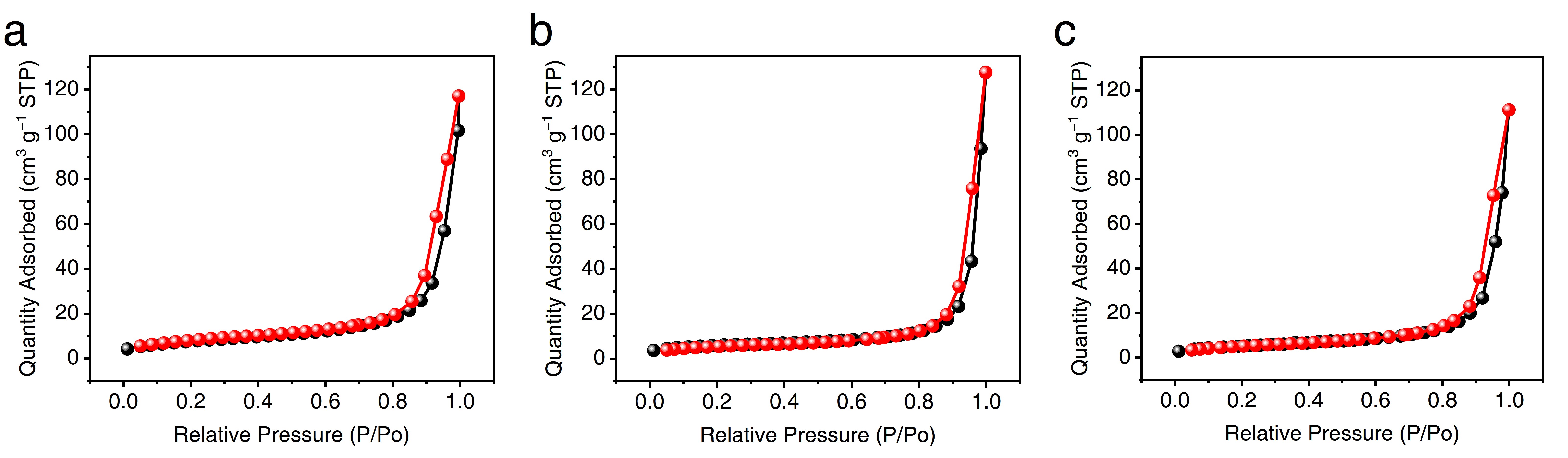


**Fig. S3** The N_2_ adsorption/desorption isotherms of **a** CoWO_4_/WO_2_, **b** CoWO_4_ and **c** WO_2_. The BET surface areas are calculated to be 28.67 m^−2^ g^−1^ for CoWO_4_/WO_2_, 26.06 m^−2^ g^−1^ for CoWO_4_, and 24.71 m^−2^ g^−1^ for WO_2_, respectively


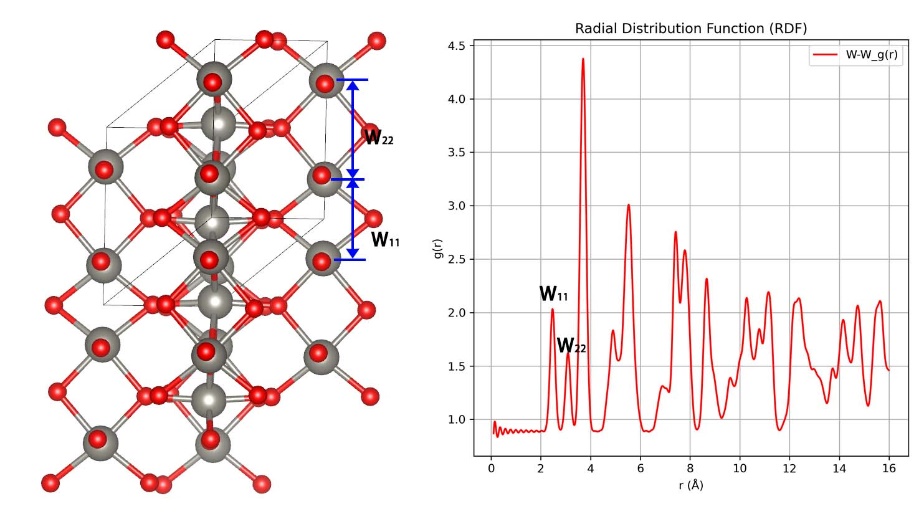


**Fig. S4** Simulated radial distribution function of W-W paths in WO_2_


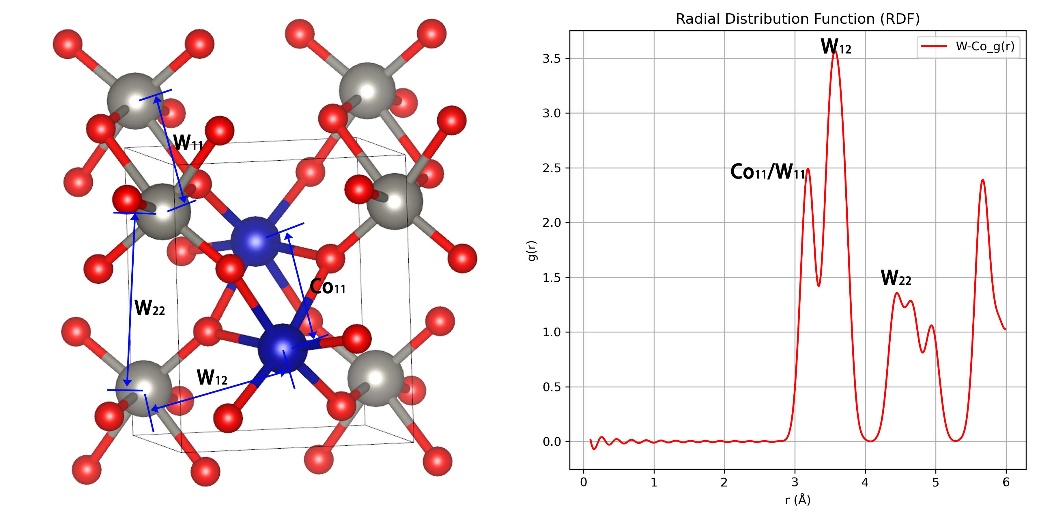


**Fig. S5** Simulated radial distribution function of M-M (M=W, Co) paths in CoWO_4_


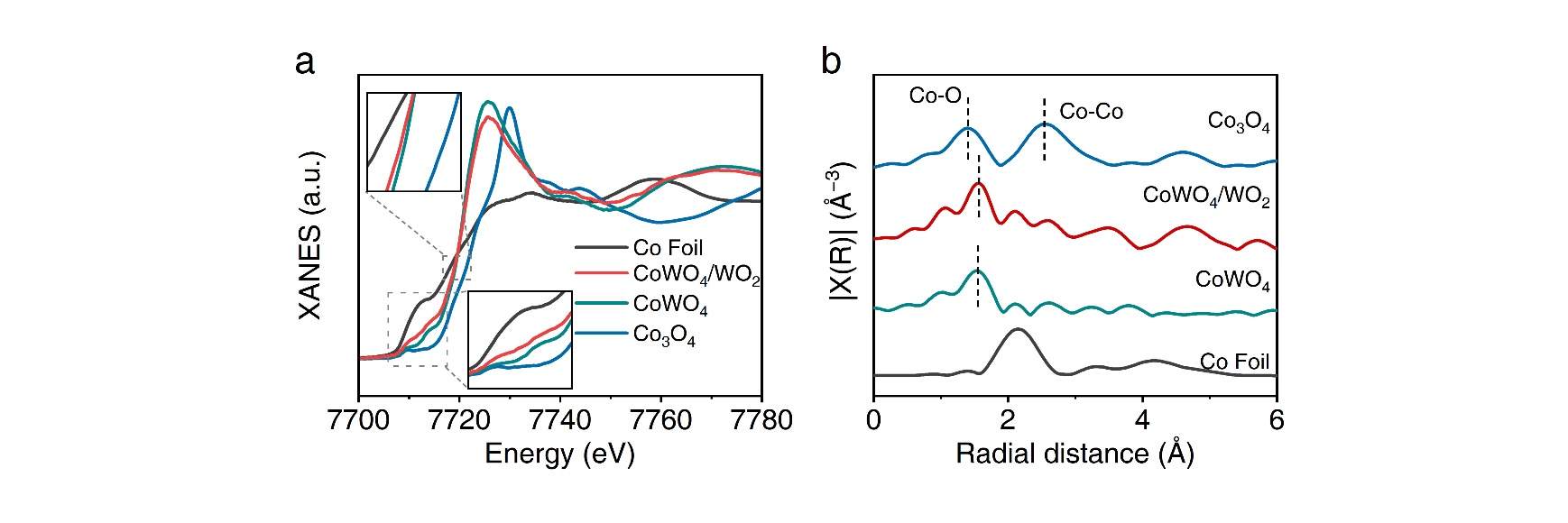


**Fig. S6** **a** XANES spectrum and **b** corresponding FT of *k*²-weighted χ(k) function for Co Foil, Co_3_O_4_, CoWO_4_, and CoWO_4_/WO_2_


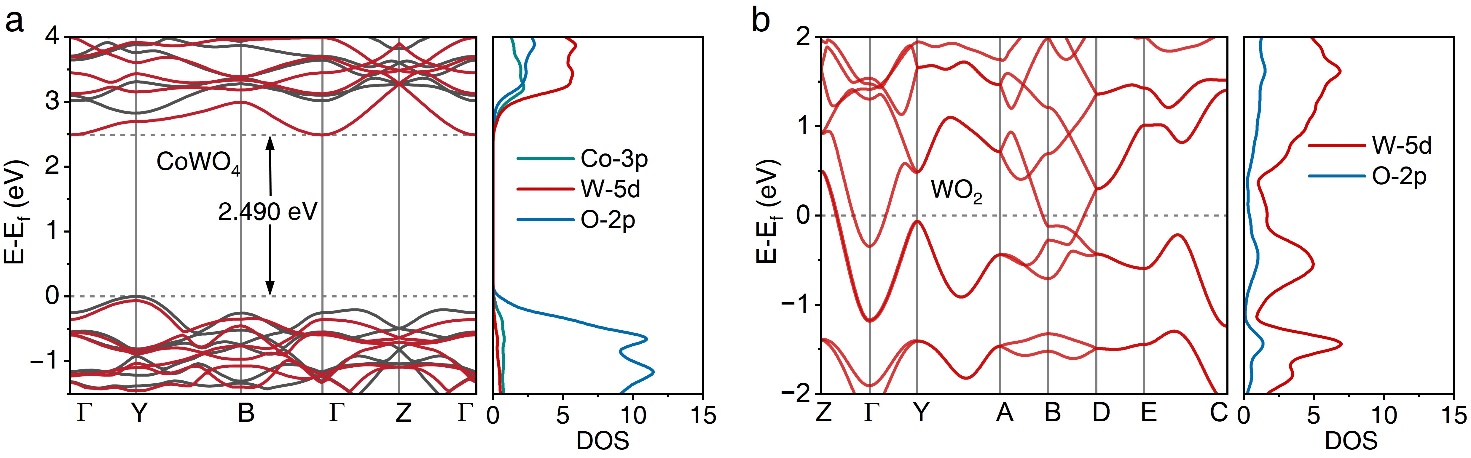


**Fig. S7** Electronic band structure and pDOS plots of **a** CoWO_4_ and **b** WO_2_


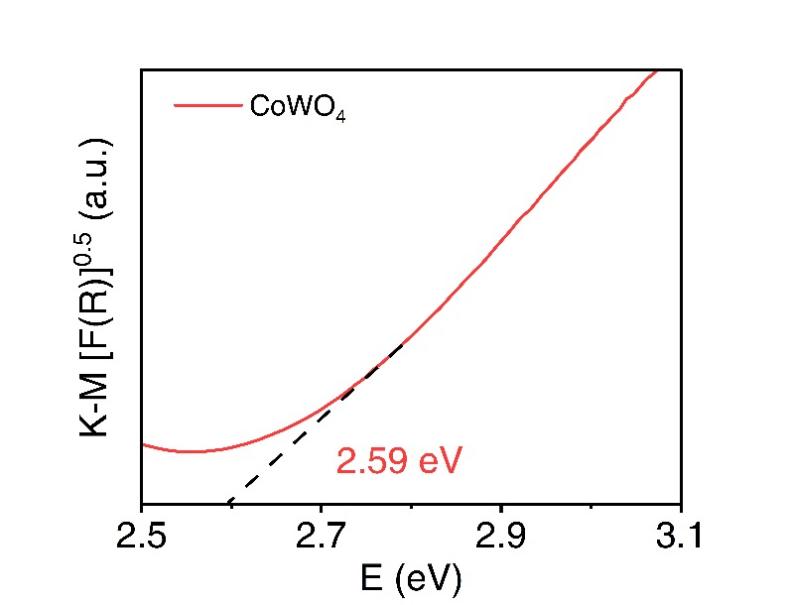


**Fig. S8** UV-vis spectrum for determining the optical bandgap of CoWO_4_


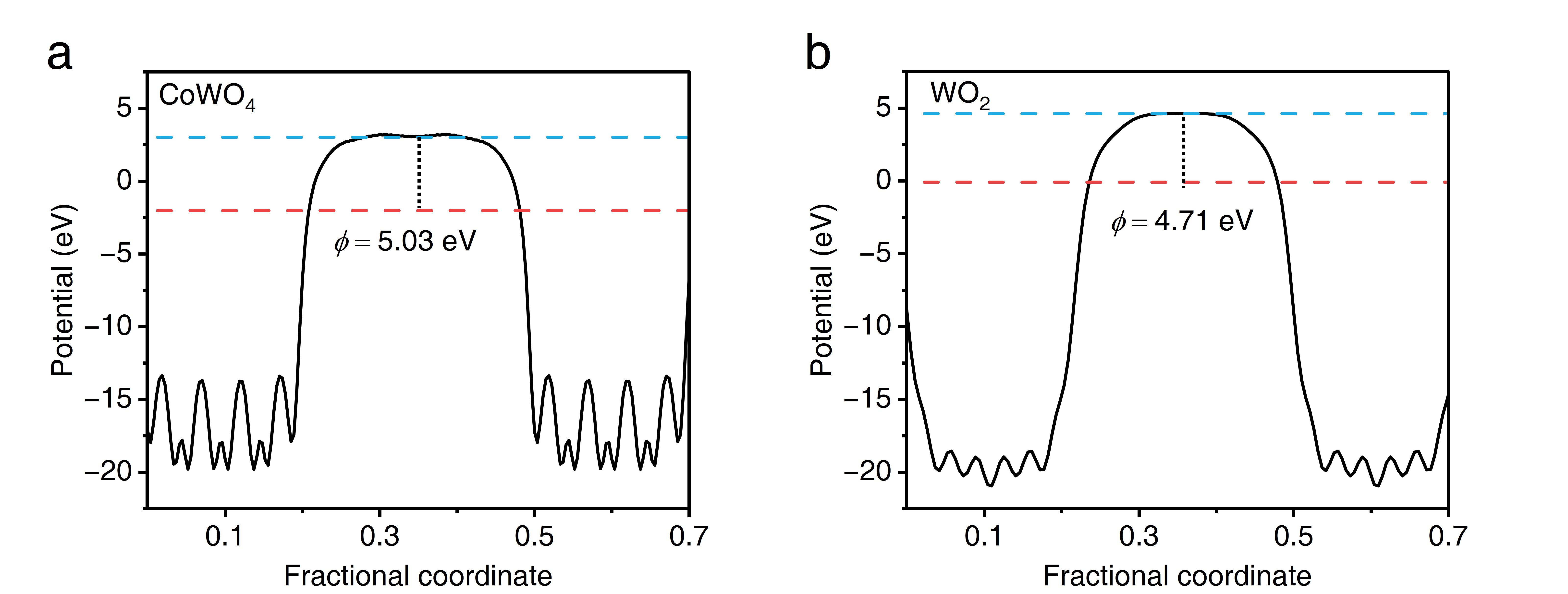


**Fig. S9** The work function (*Φ*) calculation for **a** CoWO_4_ and **b** WO_2_


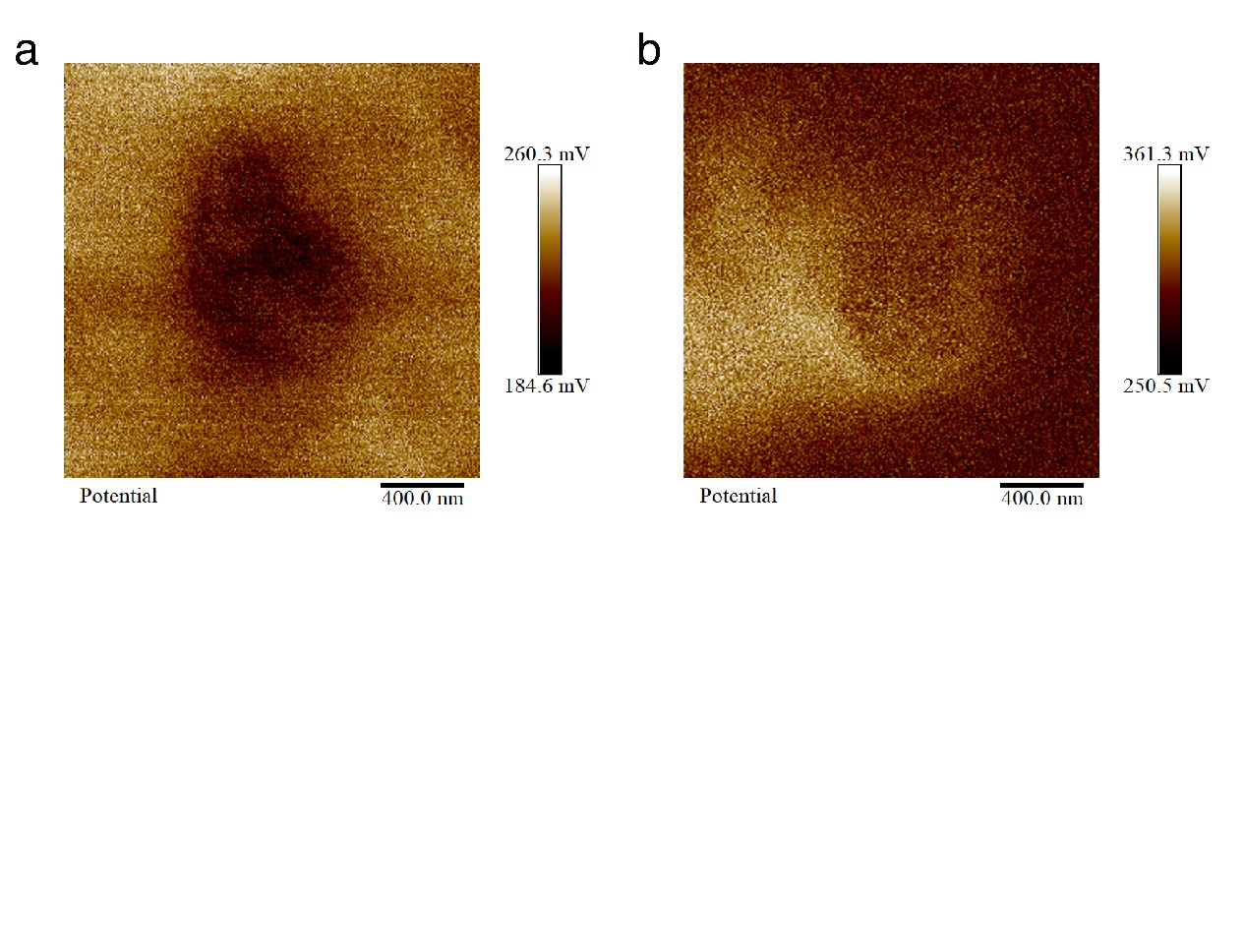


**Fig S10** KPFM images of **a** CoWO_4_ and **b** WO_2_. The work function was calculated from CPD values, calibrated against an Au reference (5.1 eV). The results show that the work function of CoWO_4_ is 5.01 eV, while the work function of WO_2_ is 4.72 eV

**
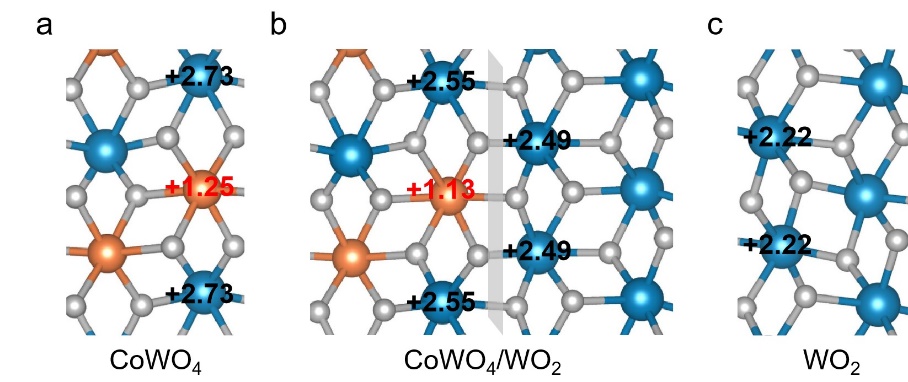
**

**Fig S11** Bader charge calculation for **a** CoWO_4_, **b** CoWO_4_/WO_2_, and **c** WO_2_. The labels on each cation is the difference in Bader charge relative to W^0^ or Co^0^. The results indicate that both Co and W in the CoWO_4_ side of the heterojunction exhibit reduced oxidation states compared to bulk CoWO_4_, while W atoms in the WO_2_ side show an increase in oxidation state due to electron loss. These findings confirm that charge transfer occurs from WO_2_ to CoWO_4_ at the interface


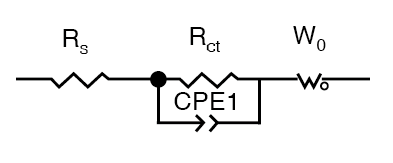


**Fig. S12** Equivalent circuit diagram for EIS curve fitting. *R_s_* represents the serial resistance of contact and electrolyte. *R_ct_* is the charge transfer resistance. *W_o_* is the Warburg impedance which reflects the diffusion impedance in electrochemical reactions


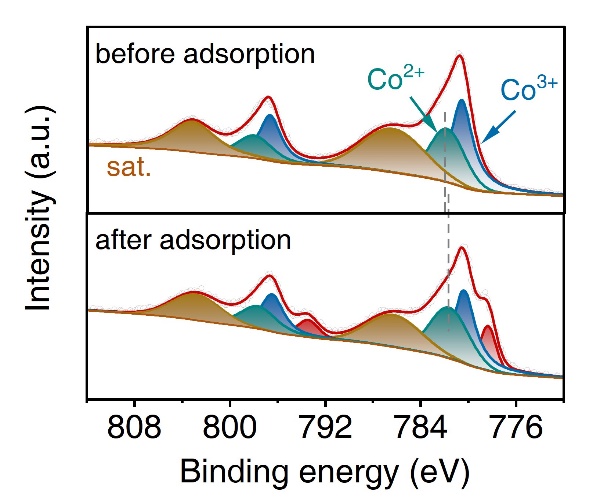


**Fig. S13** XPS spectra of Co 2p before and after the CoWO_4_/WO_2_ heterojunctions adsorb Li_2_S_4_


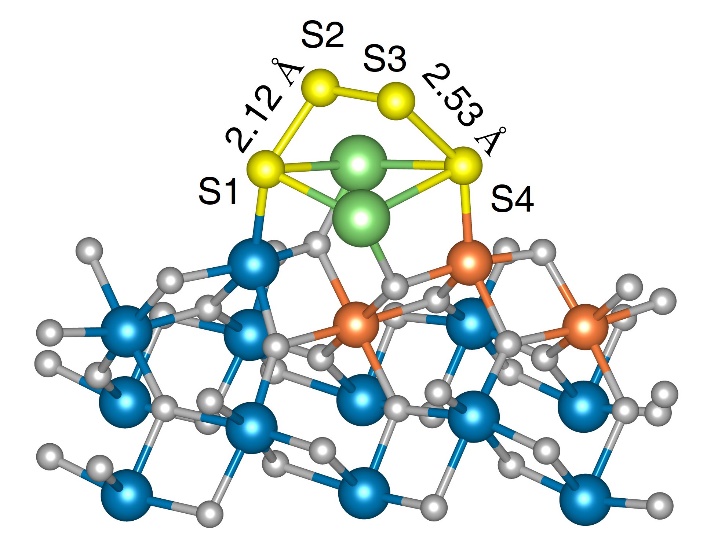


**Fig. S14** Changes in the length of S−S bonds for CoWO_4_/WO_2_-Li_2_S_4_


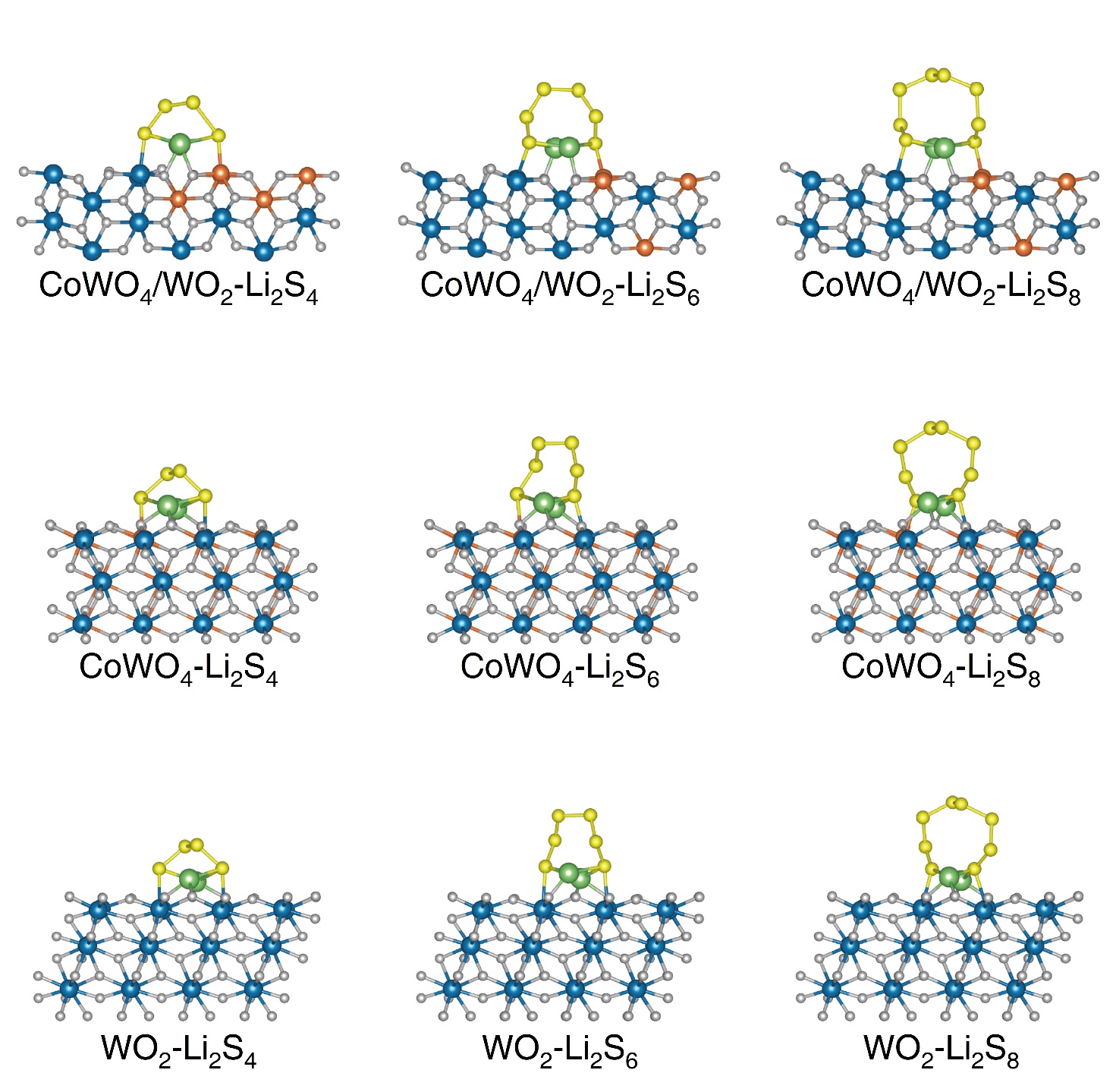


**Fig. S15** Geometric models of polysulfide molecules adsorbed on surface of CoWO_4_/WO_2_, CoWO_4_ and WO_2_


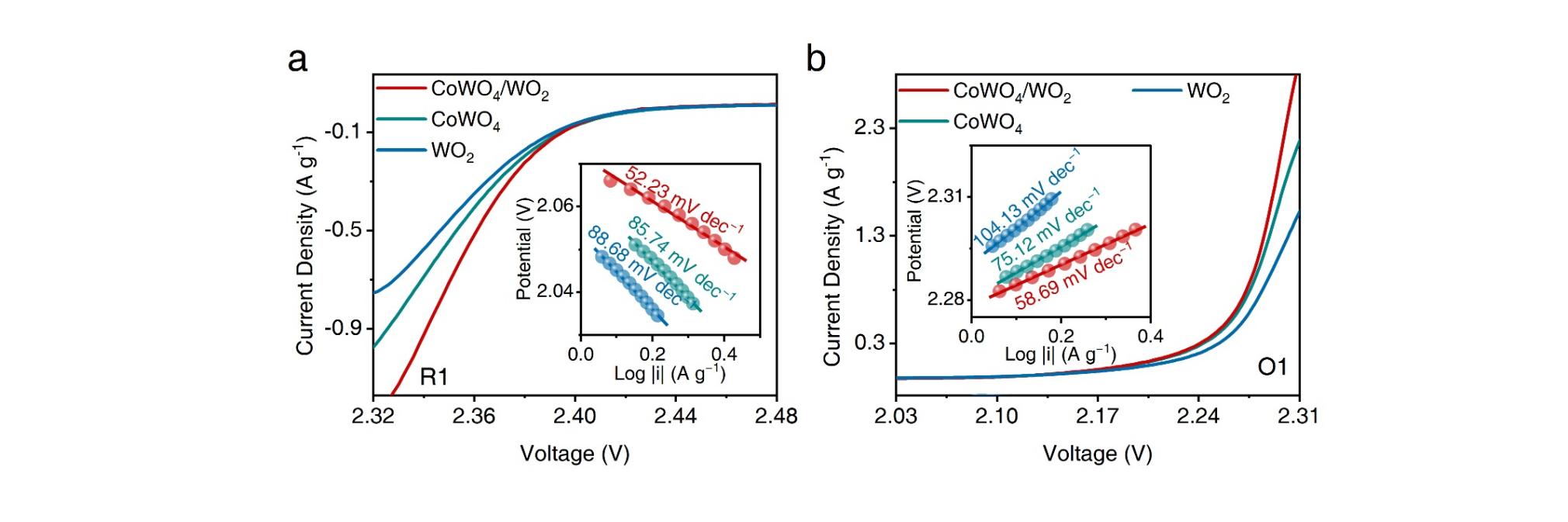


**Fig. S16** Tafel analyses of **a** peak R1 in the CV curves and **b** peak O1 in the CV curves


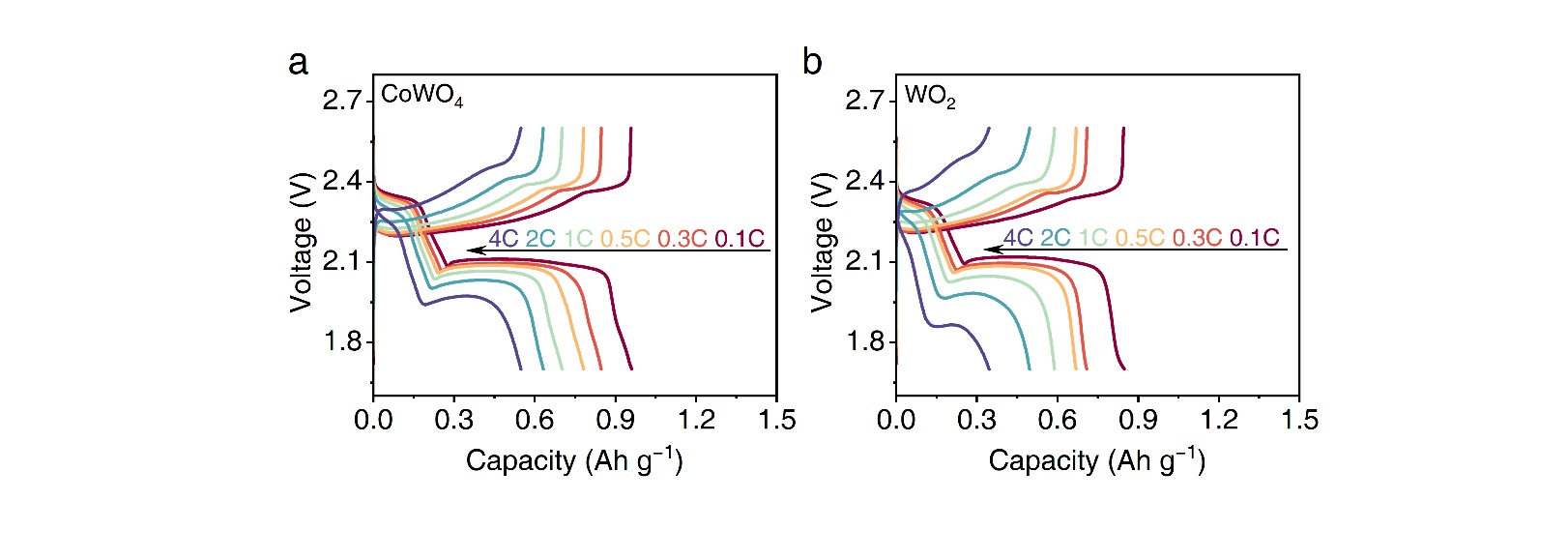


**Fig. S17** Constant current charge-discharge curves of **a** CoWO_4_ and **b** WO_2_ at different rates


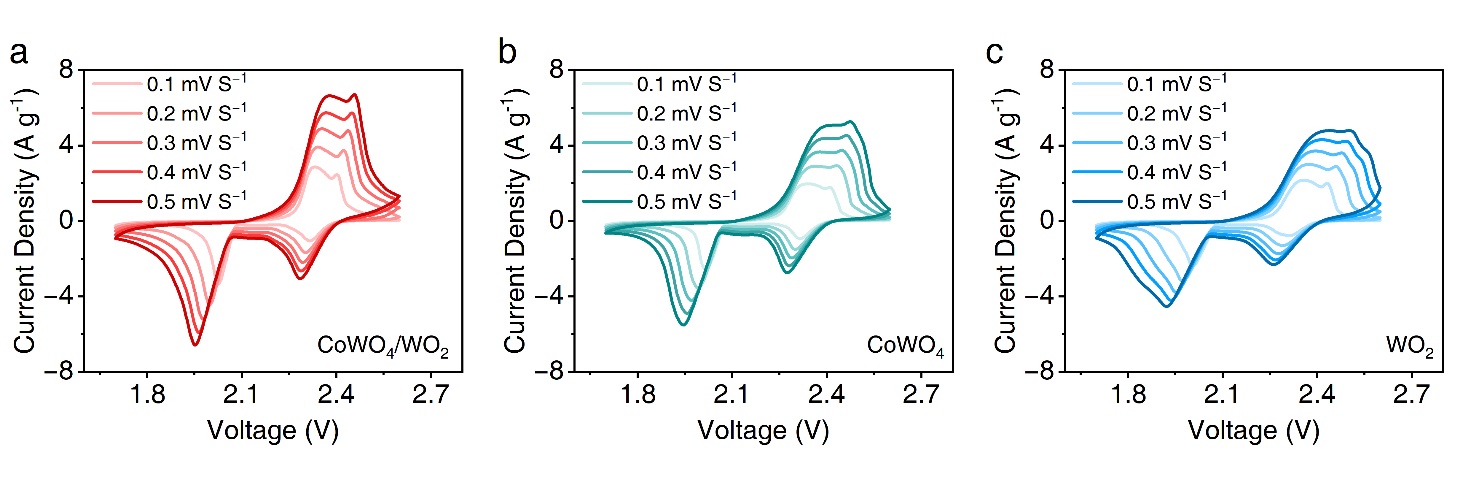


**Fig. S18** CV curves of **a** CoWO_4_/WO_2_, **b** CoWO_4_, and **c** WO_2_ were measured at 0.1, 0.2, 0.3, 0.4 and 0.5 mV S^−1^ scan rates to calculate the intrinsic kinetic rate constant ($k_{0}$)


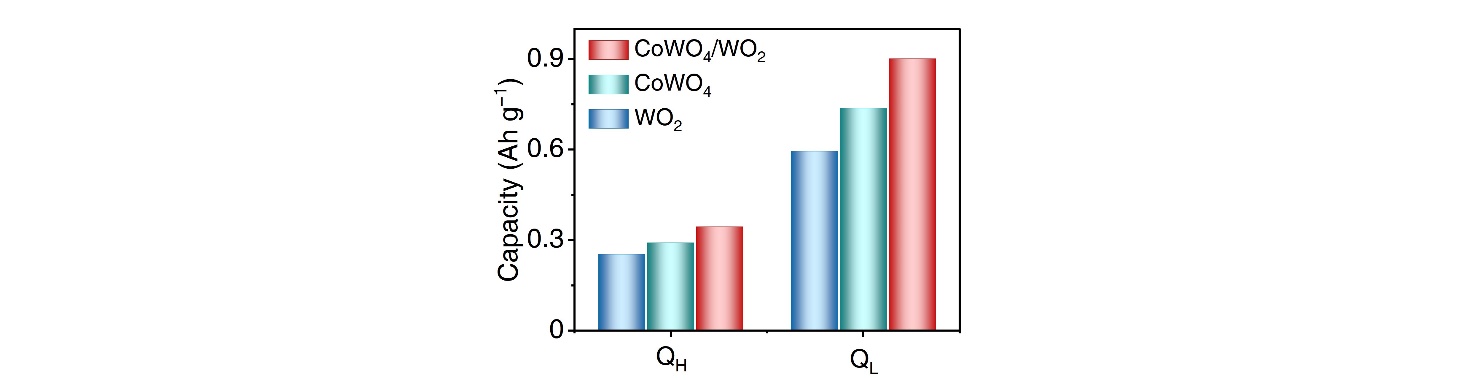


**Fig. S19** Measured Q_H_ and Q_L_ in the charge-discharge curves of Li-S batteries using CoWO_4_/WO_2_, CoWO_4_, and WO_2_ as the catalysts, respectively


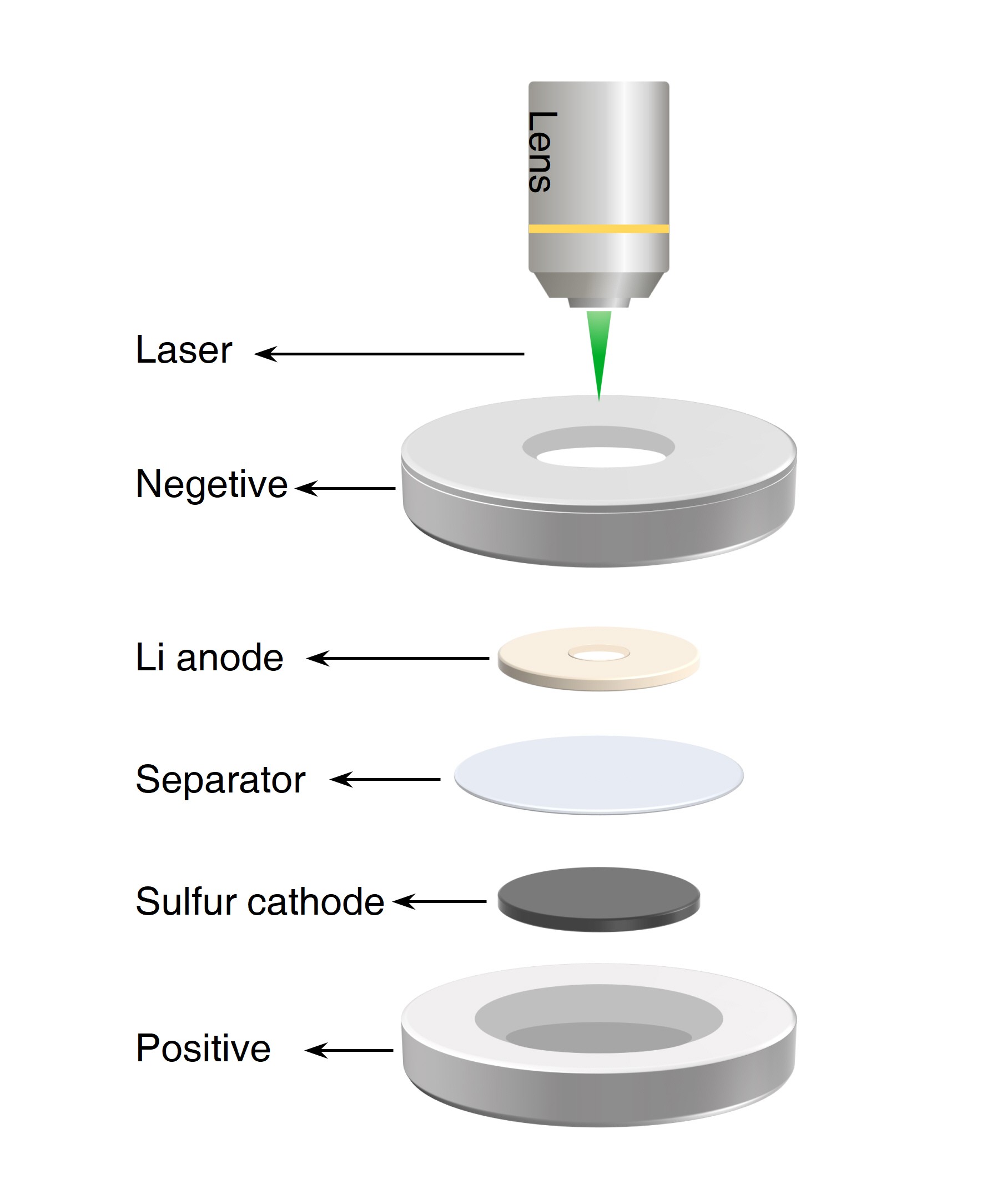


**Fig. S20** Schematic diagram of the in-situ Raman setup for Li-S batteries

**
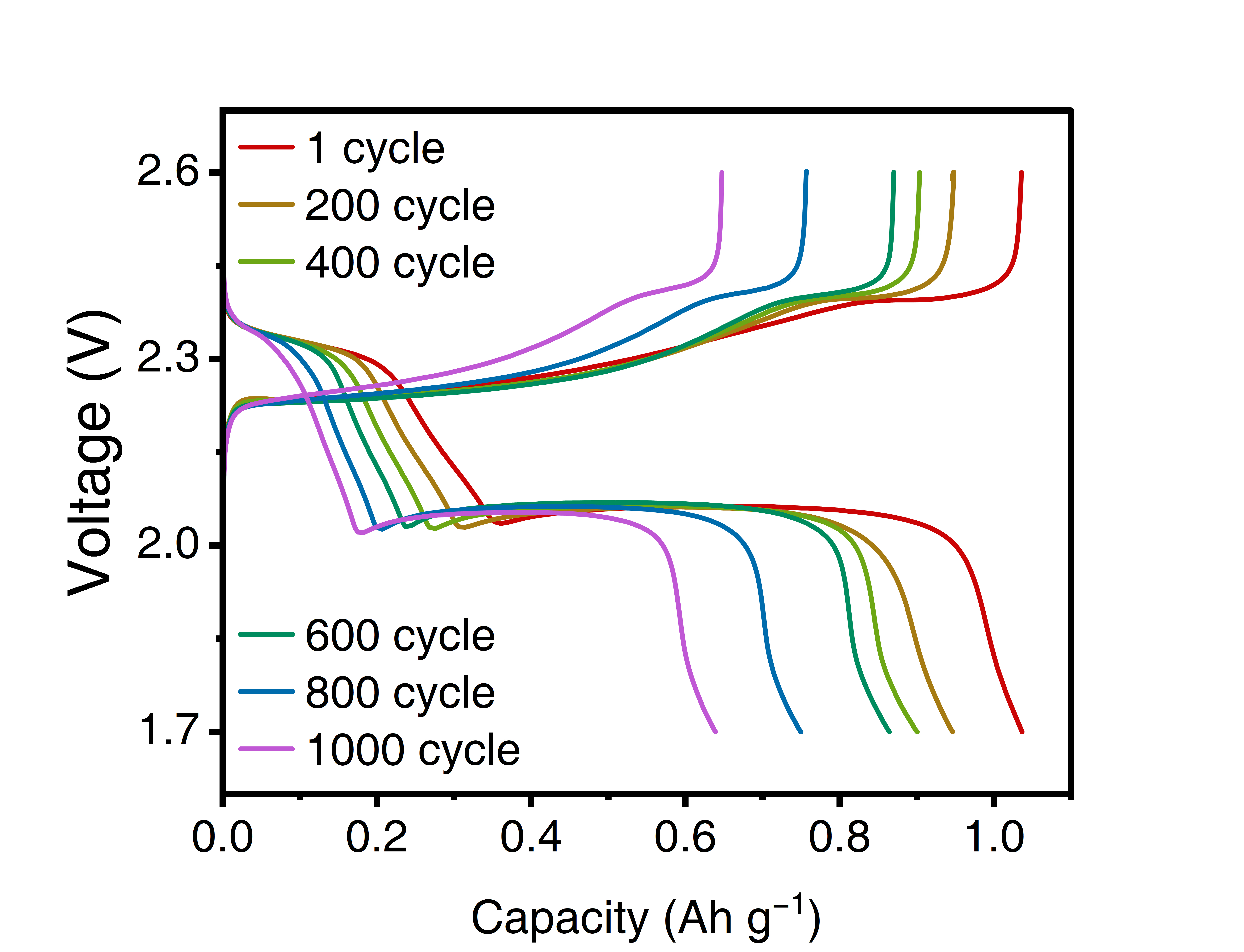
**

**Fig. S21** Charging and discharge curve diagrams of CoWO_4_/WO_2_ at the 100th and 500th circles in the long cycle

**Table S1** Elemental composition of catalysts

| Sample | Co (mol %) | W (mol %) |
| --- | --- | --- |
| CoWO_4_/WO_2_ | 38.6% | 61.4% |

**Table S2** Fitted parameters of EIS data obtained from the different catalyst-loaded cells according the equivalent circuit in Fig. S10

| Samples | Rs(Ω) | R_ct_(Ω) |
| --- | --- | --- |
| CoWO_4_/WO_2_ | 1.076 | 11.85 |
| CoWO_4_ | 1.744 | 16.07 |
| WO_2_ | 2.273 | 23.42 |

**Table S3** Beweick, M. Fleischman, and H.R. Thirsk (BFT) model and Scharifker-Hills (SH) Model: (1) two-dimensional instantaneous nucleation (2DI); (2) two-dimensional progressive nucleation (2DP); (3) three-dimensional instantaneous nucleation (3DI); (4) three-dimensional progressive nucleation (3DP)

| 2DI | $\frac{I}{I_{m}}=\left(t/t_{m}\right)\exp\left[-\frac{1}{2}\left(\frac{t^{2}-t_{m}^{2}}{t_{m}^{2}}\right)\right]$ |
| --- | --- |
| 2DP | $\frac{I}{I_{m}}=\left(t/t_{m}\right)^{2}\exp\left[-\frac{2}{3}\left(\frac{t^{3}-t_{m}^{3}}{t_{m}^{3}}\right)\right]$ |
| 3DI | $\frac{I^{2}}{I_{m}^{2}}=\frac{1.9542}{t/t_{m}}\left[1-\exp\left(-1.2564\frac{t}{t_{m}}\right)\right]^{2}$ |
| 3DP | $\frac{I^{2}}{I_{m}^{2}}=\frac{1.2254}{t/t_{m}}\left\{1-\exp\left[-2.3367\left(\frac{t}{t_{m}}\right)^{2}\right]\right\}^{2}$ |

**Table S4** Fitting parameters of Nyquist plots of Li-S full cells using the different catalysts

| Samples | Rs(Ω) | Rct(Ω) |
| --- | --- | --- |
| CoWO_4_/WO_2_ | 2.702 | 31.11 |
| CoWO_4_ | 2.698 | 45.15 |
| WO_2_ | 2.912 | 65.54 |

**Table S5** Performance comparison between CoWO_4_/WO_2_ and other materials

| Catalysts | Capacity at low rates  (mAh g^−1^) | Long-term cycling  (Cycles, Decay per cycle/Capacity Retention, Rate) | Refs. |
| --- | --- | --- | --- |
| CoWO_4_/WO_2_ | 1262 (0.1C) | 1000, 0.038%, 1C | This work |
| LaNi_0.6_Co_0.4_O_3_ | 1140.4 (0.1C) | 700, 0.08%, 1C | [S1] |
| Co-Nx | 1150 (0.2C) | 1000, 0.016%, 0.5C | [S2] |
| Co/Co_0.85_Se@NC | 1466 (0.2C) | 1000, 0.042%, 2C | [S3] |
| Co_5.47_N@NC | 1245 (0.1C) | 200, 85%, 1C | [S4] |
| CoTe_2_/Co-O-NC | 1040 (0.2C) | 500, 0.046%,0.5C | [S5] |
| Co-HTP/CG | 1137 (0.1C) | 500, 0.052%, 1C | [S6] |
| Co_7_Fe_3_/Co | 1124.9 (0.1C) | 1000, 0.046%, 1C | [S7] |
| La-deficient LaCoO_3_ | 1302 (0.1C) | 500, 0.055%, 1C | [S8] |
| Co_3_O_4_/TiO_2_-HPs | 1169 (0.2C) | 500, 0.07%, 1C | [S9] |
| CoSe_2_/Co_3_O_4_@NC-CNT | 1457 (0.1C) | 500, 0.045%, 1C | [S10] |
| S/Co-NC/WNO | 1028.5 (0.2C) | 500, 0.04%, 1C | [S11] |
| WC-WO_3_/C | 1239.6 (0.2C) | 800, 0.0058%, 2C | [S12] |
| W_2_N/Mo_2_N@MOF-C | 1631.4 (0.1C) | 980, 0.034%, 1C | [S13] |
| rGO@WO_3_ | 1410 (0.1C) | 500, 0.086%, 3C | [S14] |
| W_0.02_-Co_3_O_4_ | 1217 (0.2C) | 500, 74%, 1C | [S15] |

### Supplementary References

1. S. Hong, Q. Li, J. Li, L. Jin, L. Zhu et al., Hollow defect-rich nanofibers as sulfur hosts for lithium-sulfur batteries. ACS Appl. Mater. Interfaces **16**(27), 35063–35073 (2024). <https://doi.org/10.1021/acsami.4c05675>
2. H. Yang, L. Wang, C. Geng, Y. Zhao, Q. Li et al., Catalytic solid-state sulfur conversion confined in micropores toward superhigh coulombic efficiency lithium-sulfur batteries. Adv. Energy Mater. **14**(21), 2400249 (2024). <https://doi.org/10.1002/aenm.202400249>
3. H. Xu, Q. Jiang, K.S. Hui, S. Wang, L. Liu et al., Interfacial “double-terminal binding sites” catalysts synergistically boosting the electrocatalytic Li_2_S redox for durable lithium-sulfur batteries. ACS Nano **18**(12), 8839–8852 (2024). <https://doi.org/10.1021/acsnano.3c11903>
4. M. Li, H. Liu, Z. Cheng, J. He, H. Li et al., Atomic-level modulation of imine and cobalt enables a homogeneous Co_5.47_N catalyst for high-performance lithium-sulfur batteries. Adv. Energy Mater. **15**(21), 2405766 (2025). <https://doi.org/10.1002/aenm.202405766>
5. Z. Yang, R. Yan, J. Han, T. Wu, Q. Wu et al., Oxygen bridges of CoTe_2_/Co─O─NC enhancing adsorption-catalysis of polysulfide for stable lithium–sulfur batteries. Adv. Funct. Mater. **35**(13), 2417834 (2025). <https://doi.org/10.1002/adfm.202417834>
6. Q. Lv, Y. Sun, B. Li, C. Li, Q. Zhang et al., Metal–organic frameworks with axial cobalt–oxygen coordination modulate polysulfide redox for lithium–sulfur batteries. Adv. Energy Mater. **15**(5), 2403223 (2025). <https://doi.org/10.1002/aenm.202403223>
7. L. Sun, H. Xu, J. Xie, Y. Yuan, H. Wang et al., D-band center modulation of metallic co-incorporated Co_7_Fe3 alloy heterostructure for regulating polysulfides in highly efficient lithium-sulfur batteries. Adv. Funct. Mater. **35**(10), 2416826 (2025). <https://doi.org/10.1002/adfm.202416826>
8. Z. Bai, Z. Wang, T. Wang, Z. Wu, X. Gao et al., Cation-vacancy engineering modulated perovskite oxide for boosting electrocatalytic conversion of polysulfides. Adv. Funct. Mater. **35**(14), 2419105 (2025). <https://doi.org/10.1002/adfm.202419105>
9. H. Li, C. Chen, Y. Yan, T. Yan, C. Cheng et al., Utilizing the built-in electric field of p–n junctions to spatially propel the stepwise polysulfide conversion in lithium–sulfur batteries. Adv. Mater. **33**(51), 2105067 (2021). <https://doi.org/10.1002/adma.202105067>
10. R. Chu, T.T. Nguyen, Y. Bai, N.H. Kim, J.H. Lee, Uniformly controlled treble boundary using enriched adsorption sites and accelerated catalyst cathode for robust lithium–sulfur batteries. Adv. Energy Mater. **12**(9), 2102805 (2022). <https://doi.org/10.1002/aenm.202102805>
11. J. Zhou, Q. Qu, W. Zhang, H. Wang, D. Zhang et al., A hierarchical porous tungsten oxynitride-based nanocomposite for high-performance lithium–sulfur batteries. ACS Appl. Nano Mater. **7**(20), 23703–23711 (2024). <https://doi.org/10.1021/acsanm.4c04083>
12. Y. Zhu, S. Yang, Z. Wei, H. He, Y. Zhu et al., Revealing dynamic sulfidation of WC-WO_3_ heterogeneous nanoparticles: *in situ* formation of WS_2_ facilitates sulfur redox in Li–S battery. Carbon **232**, 119790 (2025). <https://doi.org/10.1016/j.carbon.2024.119790>
13. Y. Song, P. Tang, Y. Wang, Y. Wang, L. Bi et al., Reinforced Lewis covalent bond by twinborn nitride heterostructure for lithium-sulfur batteries. J. Energy Chem. **88**, 363–372 (2024). <https://doi.org/10.1016/j.jechem.2023.09.030>
14. L. Ni, S. Duan, H. Zhang, J. Gu, G. Zhao et al., A 3D Graphene/WO_3_ nanowire composite with enhanced capture and polysulfides conversion catalysis for high-performance Li–S batteries. Carbon **182**, 335–347 (2021). <https://doi.org/10.1016/j.carbon.2021.05.056>
15. S. Wang, R. Hu, D. Yuan, L. Zhang, C. Wu et al., Single-atomic tungsten-doped Co_3_O_4_ nanosheets for enhanced electrochemical kinetics in lithium–sulfur batteries. Carbon Energy **5**(8), e329 (2023). <https://doi.org/10.1002/cey2.329>
